# E2F1 Orchestrates Transcriptomics and Oxidative Metabolism in Wharton’s Jelly-Derived Mesenchymal Stem Cells from Growth-Restricted Infants

**DOI:** 10.1371/journal.pone.0163035

**Published:** 2016-09-15

**Authors:** Peck Yean Tan, Cheng Wei Chang, Kaibo Duan, Michael Poidinger, Kai Lyn Ng, Yap Seng Chong, Peter D. Gluckman, Walter Stünkel

**Affiliations:** 1 Singapore Institute for Clinical Sciences, Agency for Science Technology and Research (A*STAR), Singapore, Singapore; 2 Singapore Immunology Network, Agency for Science Technology and Research (A*STAR), Singapore, Singapore; 3 Department of Obstetrics and Gynaecology, Yong Loo Lin School of Medicine, National University of Singapore, Singapore, Singapore; 4 Liggins Institute, University of Auckland, Auckland, New Zealand; University of Illinois at Chicago, UNITED STATES

## Abstract

Wharton’s jelly-derived Mesenchymal Stem Cells (MSCs) isolated from newborns with intrauterine fetal growth restriction were previously shown to exert anabolic features including insulin hypersensitivity. Here, we extend these observations and demonstrate that MSCs from small for gestational age (SGA) individuals have decreased mitochondrial oxygen consumption rates. Comparing normally grown and SGA MSCs using next generation sequencing studies, we measured global transcriptomic and epigenetic profiles and identified E2F1 as an over-expressed transcription factor regulating oxidative metabolism in the SGA group. We further show that E2F1 regulates the differential transcriptome found in SGA derived MSCs and is associated with the activating histone marks H3K27ac and H3K4me3. One of the key genes regulated by E2F1 was found to be the fatty acid elongase ELOVL2, a gene involved in the endogenous synthesis of docosahexaenoic acid (DHA). Finally, we shed light on how the E2F1-ELOVL2 pathway may alter oxidative respiration in the SGA condition by contributing to the maintenance of cellular metabolic homeostasis.

## Introduction

There is a developmental component to the origin of metabolic diseases such as obesity, type 2 diabetes mellitus, and cardiovascular disease. Early studies focused on fetal growth impairment as a risk factor for later disease [1, 2]. Whereas suboptimal prenatal nutrient supply and placental dysfunction can impair fetal growth, the long-term effects of early experience that increase an individual’s metabolic risk are thought to be dependent on altered epigenetic regulation of gene expression established within a critical window of fetal development [3–5].

To provide insights into future onset of metabolic disease, we used human Wharton’s jelly-derived MSCs which are more primitive than MSCs derived from other sources [6] and retain the potential to differentiate into multiple lineages. As these MSCs originate from the umbilical cord, cellular changes in gene transcription and epigenetics may be passed onto the fetus, and thus, predispose it to later health risk. Our laboratory has shown that such MSCs of growth restricted neonates are hyper-anabolic with enhanced insulin sensitivity [7], reflecting the clinical situation of growth restricted infants who are often insulin hypersensitive [8], but become insulin resistant later in childhood [9]. The insulin-sensitive nature of MSCs derived from newborns with SGA was maintained when they were differentiated *in vitro* into mature adipocytes [10]. However, little is known about the mechanisms controlling the increased insulin sensitivity of SGA newborns and whether the SGA associated gene expression pattern is preserved in primary cells taken from SGA individuals.

Our goal is to understand the molecular basis of the altered metabolic state accompanying the insulin hypersensitivity found in growth restricted infants which is eventually replaced by insulin resistance in later childhood. There are close relationships between cellular energy expenditure, nutrient uptake and insulin sensitivity. Previous work has suggested that developmental programming is associated with changed mitochondrial function. For instance, fasting adult subjects with prior SGA condition were shown to have a decreased energy expenditure compared to a control group [11]. Resting energy expenditure was also reported to be lower in children with term SGA background [12], as well as in rodent studies demonstrating reduced cardiac muscle respiration in offspring from undernourished mothers [13].

In this study, we report the genome-wide transcriptomic and epigenomic studies utilizing MSCs from SGA and normally grown appropriate for gestational age (AGA) infants with the objective of identifying molecular signatures which may contribute to future metabolic risk in the former but not the latter group. To minimize molecular changes due to *in vitro* culture expansion, we worked with MSCs below passage 10. Our results show that MSCs taken from SGA newborns are programmed for rapid cell proliferation with concomitant decrease in the oxygen consumption rate (OCR). One key transcription factor highly expressed in the SGA-derived MSCs was found to be E2F1. This transcription factor not only suppresses OCR, but also regulates the transcript levels of a large fraction of differentially expressed genes (DEGs) in the SGA group. Amongst those, we identified ELOVL fatty acid elongase 2 (ELOVL2), which is involved in the metabolism of polyunsaturated fatty acids (PUFAs) and regulation of cellular respiratory capability possibly via docosahexaenoic acid (DHA) [14, 15].

## Materials and Methods

### Clinical populations and sample collection

Fresh umbilical cords were obtained from children born at the National University Hospital (NUH), Singapore. Prior written parental consent to participate in this study was obtained and ethical approval obtained by the Domain Specific Review Board (DSRB, # 2011/00355) of NUH.

### Assessment of fetal growth characteristics

Methods for the assessment of fetal growth in this study have been published [7, 10]. In short, SGA fetuses were identified via ultrasonographic parameters and defined as growing below the 10^th^ percentile when compared to a control reference population. Standard scans were conducted by trained ultrasonographers, using ultrasound machines (Aloka SSD- 4000, GE Voluson E8).

### Preparation and propagation of Mesenchymal Stem Cells (MSCs) from human umbilical cord Wharton’s jelly

The preparation, full characterization, nomenclature and growth conditions of the primary Mesenchymal Stem Cell (MSC) isolates used in this study have been reported [10]. WJ-MSCs from 9 SGA individuals were subsequently referred to as MSC-01, MSC-56, MSC-70, MSC-75, MSC-82, MSC-83, MSC-84, MSC-85 and MSC-86 while those from 5 AGA individuals as MSC-31, MSC-44, MSC-50, MSC-57 and MSC-60.

### Oxygen consumption rate (OCR)

Approximately 40,000 WJ-MSCs were seeded into each well of the XF24 Cell Culture Microplate (Seahorse Bioscience, 100777–004) overnight. A XFe24 Extracellular Flux Analyzer (Seahorse Bioscience) was used to measure the oxygen consumption rate (OCR). Prior to each assay run, cells were washed with XF Assay Medium Modified DMEM (Seahorse Bioscience, 102365–100) which was supplemented with 2 mM sodium pyruvate and 25 mM glucose and adjusted to pH 7.4. Drug compounds from the XF Cell Mito Stress Test Kit (Seahorse Bioscience, 101706–100) were then injected sequentially into each well in the following order with the final concentration in parentheses: Oligomycin (1 μM), FCCP (0.6 μM), followed by Antimycin A (1 μM) with Rotenone (1 μM). OCR was measured three times before and after each injection. Cells were harvested at the end of each run and protein concentration was determined using the Bradford assay (Biorad, 500–0205) for normalization across all wells.

### BrdU incorporation assay

WJ-MSCs were first cultured in normal growth media then starved in growth media containing only 1% fetal bovine serum for 24 hrs to synchronize cell growth. Following starvation, the cells were labeled with 5-bromo-2'-deoxyuridine (BrdU) using the FITC BrdU Flow Kit (BD PharmingenTM, 559619) for another 24 hrs before harvesting, fixing and staining according to the manufacturer’s protocol. The flow cytometric samples were analyzed using the FACSCanto^™^ and the FACSDiva software (BD Biosciences).

### Transient transfection

For short interfering RNA (siRNA) studies, WJ-MSC lines were transfected with 100nM siRNA (Dharmacon ON-TARGETplus SMARTpool) twice at 24h intervals, using Lipofectamine^™^ RNAiMAX Transfection Reagent (Invitrogen, 13778150) according to the manufacturer’s protocol. The siRNA sequences are listed in S1 Table. For overexpression studies, cells were transfected with 10 μg of DDK-tagged plasmid DNA from Origene (pCMV6 empty vector, PS100001 or pCMV6-ELOVL2, RC209232) using Lipofectamine^®^ LTX with Plus^™^ Reagent (Invitrogen, 15338100) according to the manufacturer’s protocol. Cells were harvested or assessed in downstream assays after 48 hrs from the second round of siRNA transfection or after 72 hrs from plasmid overexpression.

### SDS-PAGE and western blotting

Cells were lysed and protein concentration was determined using the Bradford reagent. PVDF membrane was probed with primary and secondary antibodies before developing using the ECL Plus Western Blotting Substrate (Pierce, 32132). Antibodies used are as follows: anti-E2F1 (Santa Cruz, sc-193X), anti-DDK (Origene, TA50011), anti-βactin (Sigma, A1978), ECL anti-rabbit IgG HRP linked whole antibody (from donkey) (Amersham, NA934V) and anti-mouse IgG HRP linked whole antibody (from sheep) (Amersham, NA931V).

### DHA treatment

WJ-MSCs were incubated with different concentrations of DHA (Sigma, D2534) before RNA isolation or measurement of mitochondrial OCR.

### MitoCarta2.0 Inventory

A comprehensive database, MitoCarta2.0 [16] comprising 1158 human mitochondrial genes, was used to identify DEGs which may be potentially involved in mitochondrial respiration pathways.

### Statistics

Data are generally presented as means ± S.E.M. from independent biological experiments. All comparisons between groups were assessed using a Student’s unpaired t-test unless otherwise stated, with p-value < 0.05 generally being considered as significant.

### RNA extraction and quantitative reverse transcription PCR (RT-qPCR)

Total cellular RNA from WJ-MSCs was extracted using TRIzol^®^ Reagent (Invitrogen, 15 596–026) and chloroform before purification with miRNeasy Mini Kit (Qiagen, 217004) according to the manufacturer’s instructions. RNA integrity was checked and the concentration was determined. 1 μg RNA was reverse transcribed using a High Capacity cDNA Reverse Transcription Kit (Applied Biosystems, Inc., 4368813) and cDNA produced was subjected to real-time RT-qPCR using the Power SYBR^®^ Green PCR Master Mix (Applied Biosystems, Inc., 4367659). Real-time RT-qPCR primer sequences are listed in S1 Table.

### Whole transcriptome RNA-sequencing (RNA-seq)

2 μg of total cellular RNA was depleted of ribosomal RNA [rRNA] using the Ribo-Zero^™^ Magnetic Gold Kit (Epicentre, MRZG126) as per manufacturer’s instructions. The removal efficiency was confirmed using RNA 6000 Pico Kit (Agilent Technologies, 5067–1513). Libraries were then constructed using the TruSeq Stranded Total RNA LT Kit A (Illumina, RS-122-2201) according to the manufacturer’s protocol. Adapter-ligated cDNA was amplified with 10 cycles using the PCR Primer Cocktail and PCR Master Mix provided. cDNA libraries were checked for quality and quantified using the DNA 1000 Kit. The basal and E2F1 knockdown RNA-seq libraries were sent to the Genome Institute of Singapore (GIS, Singapore) and the Beijing Genomics Institute (BGI, China), respectively, for sequencing as 76-bp paired-end reads on the Illumina HiSeq 2000 according to standard manufacturer’s procedures. Only WJ-MSCs from MSC-01, MSC-56, MSC-75, MSC-44, MSC-57 and MSC-60 were used for the RNA-seq library preparation.

### RNA-seq mapping, quantification and data analyses

Fastq files of RNA-seq were transferred into the Partek Genomic Suites (PGS, Partek). Reads were trimmed and filtered based on quality scores and mapped to Human Genome Build Hg19. Mapped reads were then quantified using GENCODE V19 transcriptome annotations. Using "read count by gene level" file output from PGS, genes with low counts were removed and only genes with more than 1 count per million (cpm) in at least three out of six samples were considered for subsequent analyses.

To identify DEGs between the basal RNA-seq from SGA (MSC-01, MSC-56, and MSC-75) and AGA (MSC-44, MSC-57 and MSC-60) groups, we performed differential analyses using the EdgeR R package according to the recommendation in the EdgeR vignette. A fold change cutoff of 1.3 and a p-value cutoff of 0.05 were applied. To identify genes whose expression was affected by E2F1 knockdown, we performed paired sample differential expression analysis on the E2F1 knockdown RNA-seq data using EdgeR following the recommendation in the EdgeR vignette. A fold change cutoff of 1.3 and a Benjamini Hochberg FDR cutoff of 0.05 were used.

### Chromatin immunoprecipitation (ChIP)

Cells were cross-linked with 1% formaldehyde before sonicating in SDS lysis buffer using the Diagenode Bioruptor. Fragmented chromatin was first pre-cleared with protein A-Sepharose 4B and rabbit IgG for 2 hrs before immunoprecipitating with fresh protein A-Sepharose 4B and antibody at 4°C overnight. Sepharose beads were washed as described previously [10] before eluting with 1% SDS followed by reverse cross-linking at 65°C overnight. Samples were finally purified using the QIAquick PCR Purification kit (Qiagen, 28106) according to the manufacturer’s protocol. Antibodies used include anti-E2F1 (Santa Cruz, sc-193X), normal rabbit IgG (Santa Cruz, sc-2027), anti-H3K27ac (Abcam, ab4729), anti-H3K4me1 (Abcam, ab8895), anti-H3K4me3 (Abcam, ab8580), anti-H3K36me3 (Abcam, ab9050) and anti-H3K27me3 (Millipore, 07–449).

### ChIP-sequencing (ChIP-seq)

ChIP-enriched DNA was first quantified with the Quant-iT^™^ PicoGreen^®^ dsDNA Assay Kit (Invitrogen, P11496) and libraries were prepared from 5 ng of ChIP DNA using the TruSeq ChIP Sample Preparation Kit Set A (Illumina, IP-202-1012) according to the manufacturer’s protocol with slight modifications. Adapter-ligated DNA was amplified with 13 cycles using the Primer Cocktail and Master Mix provided. Amplified products of appropriate sizes were excised from agarose gel and purified using the MinElute Gel Extraction Kit (Qiagen, 28604). DNA libraries were then checked for quality and quantified using the DNA 1000 Kit (Agilent Technologies, 5067–1504). The histone modification and E2F1 ChIP-seq libraries were sequenced by the Genome Institute of Singapore (GIS, Singapore) and the Beijing Genomics Institute (BGI, China), respectively. All libraries were sequenced as 36-bp single-end reads using the Illumina HiSeq 2000 according to standard manufacturer’s procedures. Only WJ-MSCs from MSC-01, MSC-56, MSC-75, MSC-44, MSC-57 and MSC-60 were used for the ChIP-seq library preparation.

### ChIP-seq mapping, peak calling and data analyses

All ChIP-seq data sets were aligned to Hg19 using Bowtie 1.1.0 [17]. We used MACS2 version 2.1.0.20150420 [18] to identify regions of ChIP-seq enrichment. BigWig tracks were generated from all libraries after normalizing to genomic input using BamCompare [19] with Signal Extraction Scaling (SES). The highest point in the track, within ±2 kb of the TSS, was then recorded as the score of E2F1 or histone mark for the corresponding gene. The score was further aggregated by group mean (SGA and AGA). For more accurate group comparisons, a scaling factor was determined and multiplied with the ChIP-seq read scores from libraries of the AGA-derived MSCs in order to minimize global bias with respect to all genes.

ChIP-seq binding strength was categorized according to the mean of ChIP-seq read score across all six cell lines and is as follows: low or no binding (score < 2), moderate binding (2 ≤ score ≤ 4) and high binding (score > 4). Tracks generated by BamCompare were uploaded to the WashU browser [20] for ChIP-seq binding intensity visualization.

### De novo motif analysis using HOMER

We overlapped E2F1 peaks identified by MACS in all six cell lines and discarded peaks which occurred in fewer than two cell lines. Next, we queried the intensity at the peak regions using BigWig generated by BamCompare to obtain the top 500 most enriched peaks. De novo motif analysis was then performed using HOMER (Hypergeometric Optimization of Motif EnRichment) [21].

### Transcription factor family motif enrichment using HOMER

We downloaded motif positions of transcription factors pre-scanned by HOMER [21] from http://bit.ly/1MYXDDJ. Using Fisher’s Exact Test, we screened for motifs which are differentially enriched between promoters of DEGs that are upregulated in AGA-derived MSCs and those upregulated in SGA-derived MSCs. A positive set consists of genes containing motif within ±2 kb of the TSS. To correct for multiple-testing for families with various motifs, we performed Bonferonni correction by multiplying the best p-value within the family with the number of motifs in the respective family, or alternatively, by using the median p-value, whichever is lesser to ensure fairness in ranking. Next, we performed another round of correction for each family by computing FDR using the Benjamini Hochberg procedure.

### Association of DEG and E2F1 binding sites from ENCODE

We downloaded the E2F1 binding sites in HeLa-S3 and MCF-7 cell lines from ENCODE. We then performed Fisher's Exact Test to determine the extent of preferential E2F1 binding within ±2 kb of the TSS between DEGs up- and downregulated in SGA-derived MSCs.

### Gene Ontology analysis

DEGs between SGA and AGA groups from the basal RNA-seq expression datasets were loaded into the MetaCore portal (GeneGO, a Thomson Reuters business) to identify for enriched cellular processes.

### Accession number

All library data were deposited into the GEO repository (GEO # GSE77260).

## Results

### Phenotypic and transcriptomic differences between SGA- and AGA-derived MSCs

**Fig 1:**
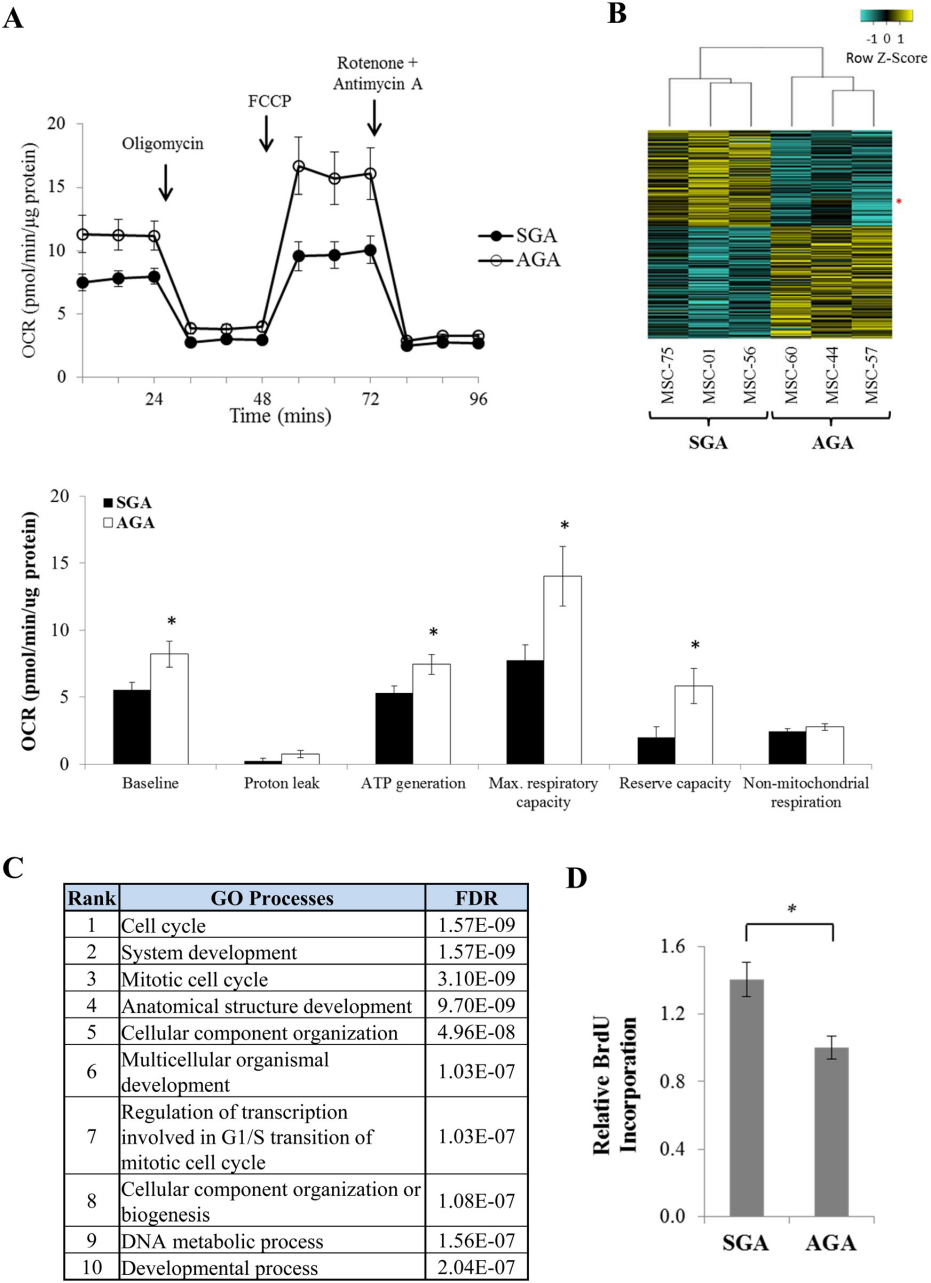
Mitochondrial oxygen consumption is repressed and cell cycle gene expression is increased in MSCs isolated from SGA newborns. (A) Basal and drug-stimulated mitochondrial oxygen consumption rates (OCR) between 9 SGA and 5 AGA lines as measured by the Seahorse XF analyser. Drugs which affect cell respiration were added in the following order: Oligomycin (ATP synthase inhibitor), FCCP (uncoupler), followed by Rotenone and Antimycin A (inhibitors of respiratory complexes I and III respectively). Top: Closed and open circles represent the OCR traces as expressed in pmol O_2_ per min normalized against protein amount upon drug treatments across time in SGA and AGA lines, respectively. Bottom: Barchart indicating the OCR measurements in terms of pmol O_2_/min/μg protein for various parameters. The data represent mean ± SEM of at least 3 independent experiments, * p < 0.05 where p-value was calculated using an unpaired t-test. (B) Heatmap illustrating basal whole transcriptomic RNA-seq expression differences of coding transcripts between 3 representative pairs of SGA (MSC-01, MSC-56 and MSC-75) and AGA lines (MSC-44, MSC-57 and MSC-60). Yellow and blue bars represent up- and downregulation of gene expression between SGA and AGA groups respectively (≥ 1.3-fold, p < 0.05). The red asterisk indicates the expression of E2F1 transcripts across all cell lines. (C) Metacore GO of DEGs between SGA and AGA lines obtained from basal RNA-seq. Table shows the top ten biological processes ranked by FDR. (D) Bars showing the fold difference of BrdU-stained cells for 9 SGA lines relative to 5 AGA lines. Results are represented as the mean ± SEM of at least 3 independent experiments, * p < 0.05 where p-value was calculated using an unpaired t-test.

Multipotent WJ-MSCs have the ability to differentiate into multiple lineages which are highly relevant to the development of metabolic diseases. Molecular signatures favoring poor health outcomes may be determined prenatally in SGA babies upon unfavorable perinatal insults [5, 22], making the readily obtainable WJ-MSCs a valuable model in studying potential biomarkers impacting future health status. We first assessed whether there were phenotypic differences in key metabolic cellular measures such as mitochondrial oxygen consumption rates (OCR) between MSC lines from 9 SGA and 5 AGA neonates. Our results show that SGA-derived MSCs generally have a significantly lower OCR readout compared to their AGA counterparts (Fig 1A), indicative of differences in energy metabolism. In an attempt to identify gene expression differences that might explain this finding, we performed RNA-seq on 6 representative MSC lines under normal growing cell culture conditions. Overall, we found 388 genes that were differentially expressed between the SGA and AGA groups, with 180 upregulated genes and 208 downregulated genes in the former group (Fig 1B). 28 of these differentially expressed genes (DEGs) were randomly selected for validation using RT-qPCR, where we observed a good correlation between the expression profiles from RNA-seq and RT-qPCR (S1 Fig). Pathway analysis revealed that the DEGs identified are significantly overrepresented in cell cycle as well as developmental processes (Fig 1C). The primary human MSC isolates established from SGA neonates indeed possess a greater proportion of BrdU-stained cells (Fig 1D). This is in concordance with an earlier study which reported that MSCs derived from growth restricted neonates have increased cell proliferation rates [7], and enhanced proliferation growth is usually accompanied with metabolic changes [23].

### E2F1 is overexpressed in MSCs isolated from SGA cells and alters the mitochondrial oxygen consumption rate

**Fig 2:**
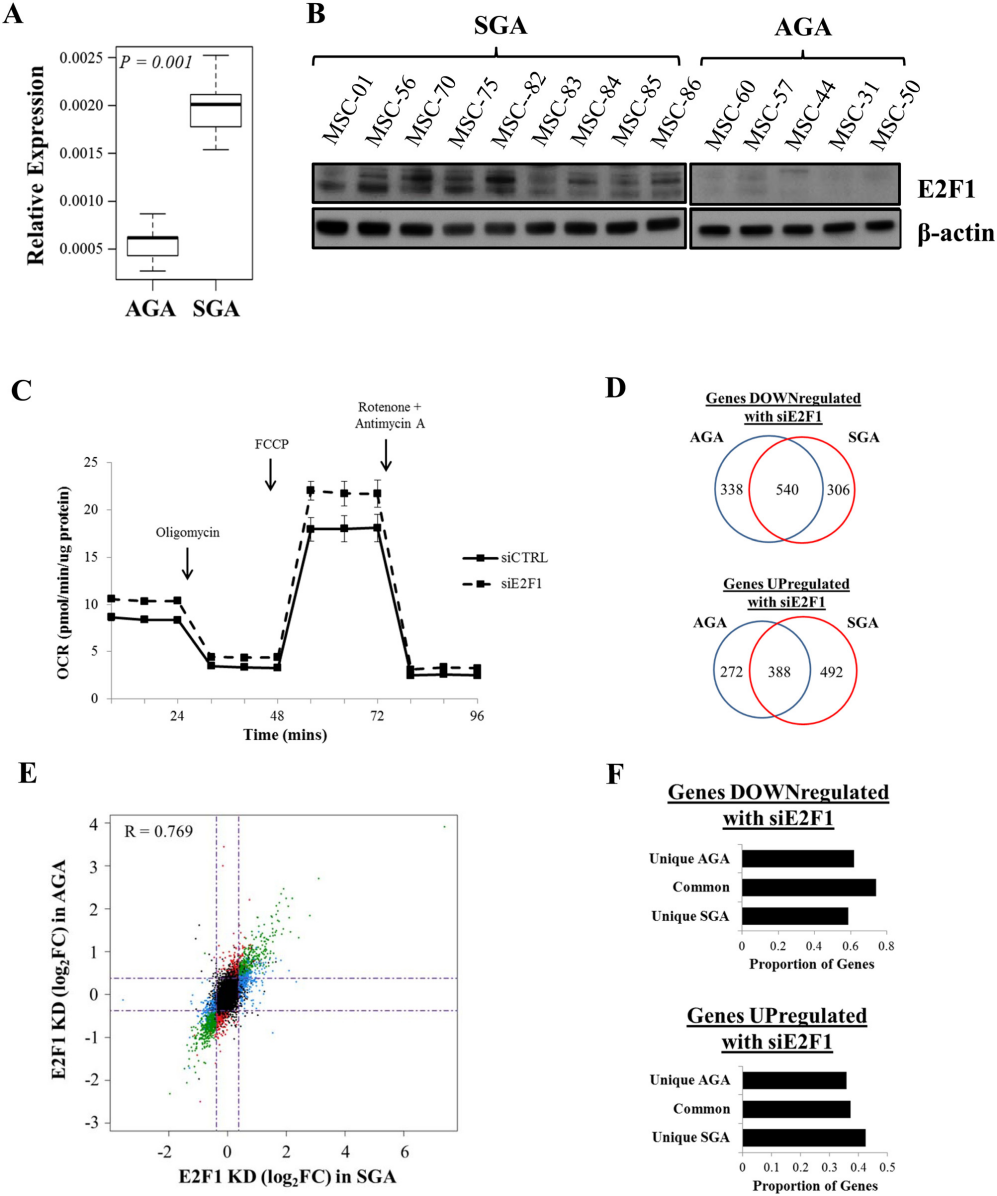
E2F1 is over-expressed in Wharton’s jelly MSCs from SGA background. (A) Boxplot illustrating the relative E2F1 gene expression in 9 SGA and 5 AGA lines. Total mRNA expression was quantified by real-time RT-qPCR. E2F1 expression levels were normalized against those of β-actin. The data represent mean ± SEM of at least 3 independent experiments. The p-value was calculated using an unpaired t-test. (B) E2F1 and β-actin protein expression levels in 9 SGA- and 5 AGA-isolated MSCs. (C) Basal and drug-induced mitochondrial OCR in the MSCs was measured after transient transfection. Solid and dotted lines refer to the OCR traces before and after E2F1 depletion, respectively in the 6 representative MSC lines (MSC-01, MSC-56, MSC-75, MSC-44, MSC-57 and MSC-60). Results represent mean ± SEM of at least 3 independent experiments. (D) Venn diagrams depicting the number of genes affected by E2F1 depletion, either unique to or common between SGA and AGA groups. Top and bottom panels show down- and upregulated genes upon siE2F1 treatment from RNA-seq, respectively. (E) Scatterplot correlating gene expression upon E2F1 suppression in SGA and AGA lines. Dotted lines indicate the log2-fold change of 0.38 approximately for both axes. Blue, red and green dots represent genes which were significantly affected in the presence of E2F1 knockdown in SGA only, AGA only and both groups, respectively. Black dots represent the remaining genes expressed in the transfected cell lines. Linear relationship was determined by a Pearson correlation coefficient (R). (F) Barcharts showing the proportion of E2F1 up- (top) and downregulated (bottom) genes which are occupied by E2F1 at gene TSS exclusively in SGA or AGA-derived MSCs or in both groups of cell lines.

Previous studies have highlighted the importance of cell cycle regulators in triggering an adaptive metabolic switch to meet higher energy demands [23]. Notably, E2F1, which is a well-characterized ‘master regulator’ of cell cycle progression, was expressed at higher transcript (Figs 1B and 2A) and protein (Fig 2B) levels in SGA-derived MSCs when we examined a panel of 14 primary cell lines. E2F1 depletion induced by siRNA transfection led to increase in mitochondrial oxygen consumption (Fig 2C), and this is in agreement with our findings from basal AGA-derived MSCs (Fig 1A) that have shown correspondingly lower expression of E2F1.

Given that E2F1 is pivotal in governing the expression of a wide repertoire of genes, we coupled E2F1 knockdown with RNA-seq to determine its downstream targets. Overall, we found 846 repressed genes upon E2F1 depletion in SGA-derived MSCs, with 540 of them also being down-regulated in AGA-derived MSCs (Fig 2D, top). We note that the highly efficient siE2F1 transfection condition (S2 fig) could have contributed to the huge disparity between the total number of DEGs (Fig 1B) and E2F1-regulated genes (Fig 2D) as difference in E2F1 expression between SGA and AGA-derived cells was approximately 2.8-fold while that before and after siE2F1 introduction was greater than 5-fold. Reduced E2F1 expression also concomitantly resulted in increased expression of 388 genes in both SGA and AGA-established MSCs (Fig 2D, bottom). Strikingly, we observed more than 35% of genes to be significantly affected by siE2F1 in only one group of MSCs but not in the other (Fig 2D). For instance, siE2F1 resulted in the repression of 306 out of 846 genes (36.2%) in SGA-derived MSCs, but not in the AGA-derived counterparts, whereas 338 out of 878 genes had diminished expression levels selectively in the latter group (38.5%, Fig 2D, top). In addition, we were able to identify 492 out of 880 genes (55.9%) and 272 out of 660 genes (41.2%) with elevated expression levels exclusively in SGA- and AGA-derived MSCs, respectively. This prompted us to explore if E2F1 could have disparate impact in regulating these genes between both groups of MSCs. To address this, we plotted the E2F1 knockdown effects on all expressed genes in both types of MSCs and found that there was a good correlation with r = 0.769 (Fig 2E). Hence, we conclude that the genes affected by siE2F1 earlier appeared to be unique to one group (Fig 2D) due to the stringent statistical cut-off used for knockdown analyses. To further identify direct E2F1 gene targets, we generated E2F1 ChIP-seq libraries to uncover genome-wide locations of E2F1 occupancy in our primary MSC isolates. Previous studies carried out in other cell lines have shown that E2F1 is typically located within the gene promoter regions [24, 25]. Similarly, we also found predominant recruitment of E2F1 (> 70% of E2F1 binding) to gene promoters (S3A Fig) with E2F1 mainly residing around the gene TSS (S3B Fig). By associating E2F1 binding with siE2F1 RNA-seq datasets, we found that even though E2F1 possesses a dual regulatory role, it acts more as a transcriptional activator than repressor (Fig 2F).

### Significant E2F1 enrichment at DEGs with enhanced expression in SGA

**Fig 3:**
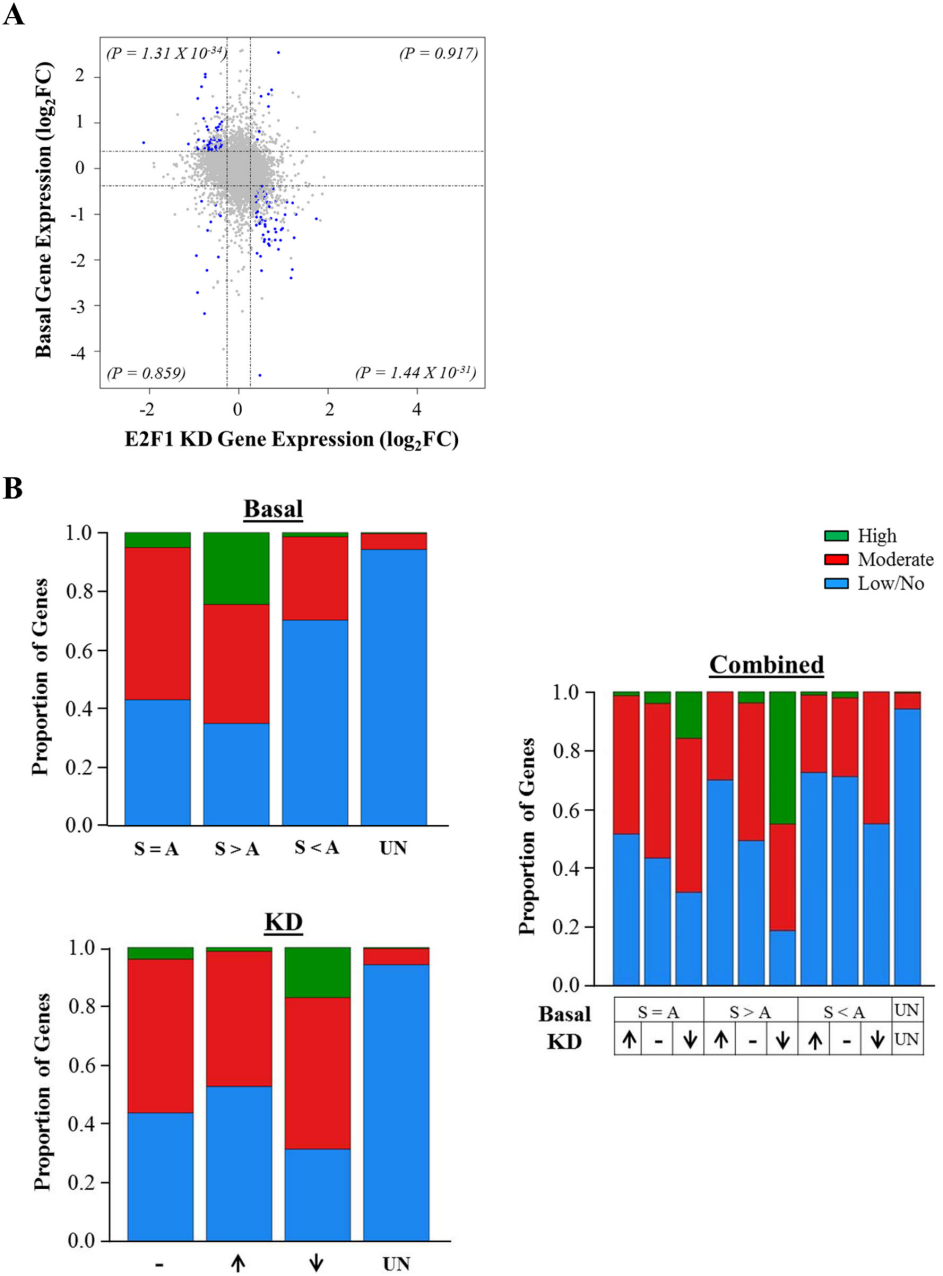
E2F1 directly regulates DEGs overexpressed in SGA-derived MSCs. (A) Scatterplot illustrates log2-fold change of basal gene expression between 3 SGA and 3 AGA lines against log2-fold change of gene expression upon siE2F1 treatment in the same MSC lines. Dotted lines indicate the log2-fold change of 0.38 approximately for both axes. Each blue dot represents a gene which was identified as being both a DEG and a E2F1 target gene (p-values met) whereas the grey dots represent the remaining genes expressed in the MSCs (most of which did not pass the p-value cutoff). Fisher’s exact test was used to determine the significance for the number of genes (blue dots) in each quadrant and the p-values are shown in parentheses. (B) Stacked barcharts showing the proportion of gencode V19 gene promoters with varying degrees of E2F1 binding where green, red and blue bars represent high, moderate and low or negligible E2F1 binding, respectively. Categories of “S > A”, “S < A”, “S = A” and “UN” represent DEGs with higher basal expression in SGA, DEGs with higher basal expression in AGA, expressed genes excluding DEGs and unexpressed genes, respectively. Categories of “↓”, “↑” and “-” represent genes whose expressions were downregulated, upregulated or remained unchanged upon siE2F1 treatment, respectively. The absolute number of genes in each subcategory is shown in S4 Fig.

From our transcriptomic analyses, we noticed that the expression of approximately 50% of DEGs was significantly affected by E2F1 down-regulation. Since E2F1 expression was found to be substantially higher in SGA-established MSCs, we then asked whether the difference in E2F1 levels could contribute to the observed DEG expression pattern. We compared the basal and E2F1 depletion expression profiles and found that a significant proportion of DEGs that are upregulated in the SGA-derived MSCs were down-regulated in the presence of siE2F1 (Fig 3A, p = 1.31 X 10^−34^). Conversely, DEGs which are down-regulated in the SGA-derived MSCs tended to have elevated expression upon E2F1 knockdown (p = 1.44 X 10^−31^). This, again, supports the binary transcriptional role of E2F1 in MSCs from Wharton’s jelly and strongly suggests that E2F1 is driving a significant proportion of the DEG expression.

To further dissect the importance of E2F1’s transcriptional activity, we segregated the E2F1 ChIP-seq sites into high, medium and low or negligible binding intensity and associated them with the gene expression profiles. The number of genes for each category is listed in the S4 Fig. As shown in Fig 3B, a large proportion of genes (65%) with higher basal expression in SGA-derived MSCs exert medium to high E2F1 binding strength compared to only 30% for those with lower basal expression (p<0.001, Fisher’s Exact Test). In addition, we also observed a larger percentage of E2F1-activated genes (69%) with stronger E2F1 recruitment compared to E2F1-repressed genes (47%, (p<0.001, Fisher’s Exact Test). Interestingly, upon integrating all genomic datasets, we found further enrichment of E2F1 at E2F1-activated genes that are simultaneously expressed at higher amounts in the SGA-established MSC lines. Approximately 81% of such genes are at least moderately occupied by E2F1.

### Active histone H3K27ac and H3K4me3 marks are enriched at genes upregulated in SGA-derived MSCs

**Fig 4:**
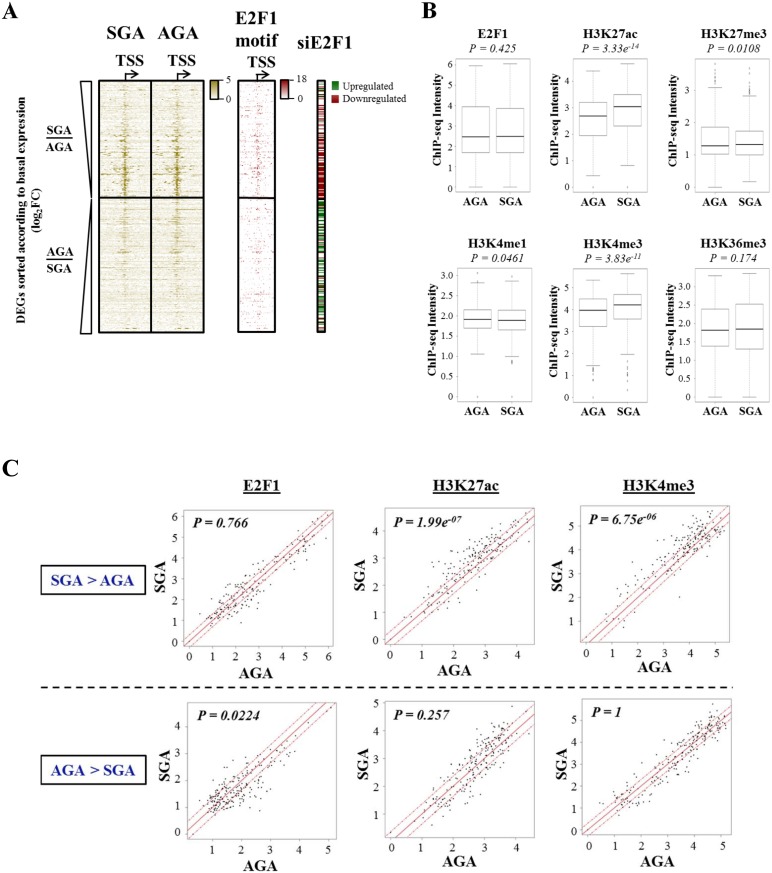
Active H3K27ac and H3K4me3 marks, but not E2F1, are enriched in SGA-derived MSCs at promoters of upregulated DEGs. (A) Heatmaps illustrating E2F1 ChIP-seq binding (left), E2F1 de novo motif enrichment (middle) and siE2F1 RNA-seq gene expression modulations (right) for all DEGs sorted according to the basal RNA-seq fold change expression difference between both groups of MSCs with SGA-upregulated genes on top followed by AGA-upregulated genes below. For the motif enrichment heatmap, the color intensity corresponds to the highest scoring motif within a 100bp window. For both binding and motif enrichments, signal intensities were plotted around the TSS ±5 kb of each DEG. (B) Boxplots comparing ChIP-seq intensities of E2F1 and five histone modifications (H3K27ac, H3K27me3, H3K4me1, H3K4me3 and H3K36me3) in MSCs established from SGA and AGA neonates. The p-values were calculated using a Wilcoxon’s test. (C) Scatterplots illustrating ChIP-seq intensities of E2F1 (left), H3K27ac (middle) and H3K4me3 (right) between SGA and AGA-derived MSCs at both groups of DEGs. Top: DEGs with higher basal gene expression in SGA-derived MSCs; bottom: DEGs with higher basal gene expression in AGA-derived MSCs. The p-values were calculated using a two-tailed binomial test.

Appropriate temporal and spatial recruitment of transcription factors and chromatin modifiers is necessary for precise modulation of gene expression. To date, several predictive models have been constructed to examine the intricate relationship between transcription factor binding, histone modifications and mRNA abundance [26, 27]. In general, among the DEGs identified, we noticed there was minimal E2F1 binding at genes that are upregulated in the AGA-derived MSCs (Fig 4A, left). However at the TSS of genes that are upregulated in the SGA counterparts, E2F1 exhibited either strong or weak binding which likely suggests direct or indirect occupancy, respectively (Fig 4A, left). Besides their promoter containing consensus E2F1 motif (Fig 4A, middle) as found by de novo motif analysis of our E2F1 ChIP-seq libraries (S5 Fig), these DEGs with higher E2F1 binding are also largely involved in cell cycle regulation (S6 Fig). We further used HOMER to identify differentially enriched transcription factor family motifs between the two groups of DEGs and found that the E2F family motif appeared as the most significant hit (S7A Fig). In addition, by using publicly available E2F1 ChIP-seq data sets from the ENCODE project, we also managed to show that E2F1 indeed binds to a greater proportion of SGA-upregulated genes compared to AGA-upregulated genes (S7B Fig). These findings corroborated our hypothesis that E2F1 plays a predominant role at DEGs with enhanced expression in SGA-derived MSCs. Since E2F1 also mediates phenotypic changes in MSCs, this prompted us to further investigate the direct transcriptional impact of E2F1 on the set of genes with higher basal expression in SGA-established MSCs.

At the promoter of SGA-upregulated genes, we found no significant difference in E2F1 binding between SGA and AGA-derived MSCs (Fig 4B and 4C), suggesting that the differential gene expression pattern observed was unlikely due to unequal E2F1 occupancy. However, from the study of the ChIP-seq of active and repressive histone marks, we noticed differential H3K27ac and H3K4me3 modifications at these genes (Fig 4B and 4C). The ChIP-seq intensities of H3K27ac and H3K4me3, which are both active histone marks, were significantly higher (p < 0.001) in the SGA-derived MSCs compared to the AGA counterparts and this is in accord with the observation that these genes were also expressed to a greater extent in the SGA-derived MSCs. In contrast, genes which were downregulated in SGA-derived MSCs had similar E2F1, H3K27ac and H3K4me3 levels generally (Fig 4C). By further splitting the SGA-upregulated genes into E2F1-bound and -unbound, we observed that only genes occupied by E2F1 at the promoter have correspondingly higher H3K27ac and H3K4me3 modifications in SGA-derived MSCs compared to AGA counterparts (S8 Fig). All in all, we postulate that E2F1 recruitment is potentially important in priming the promoter of SGA-upregulated genes for specific histone modifications (H3K27ac and H3K4me3).

### ELOVL2 is a novel E2F1-activated gene upregulated in SGA-derived MSCs and associated with histone H3K27ac and H3K4me3 marks

**Fig 5:**
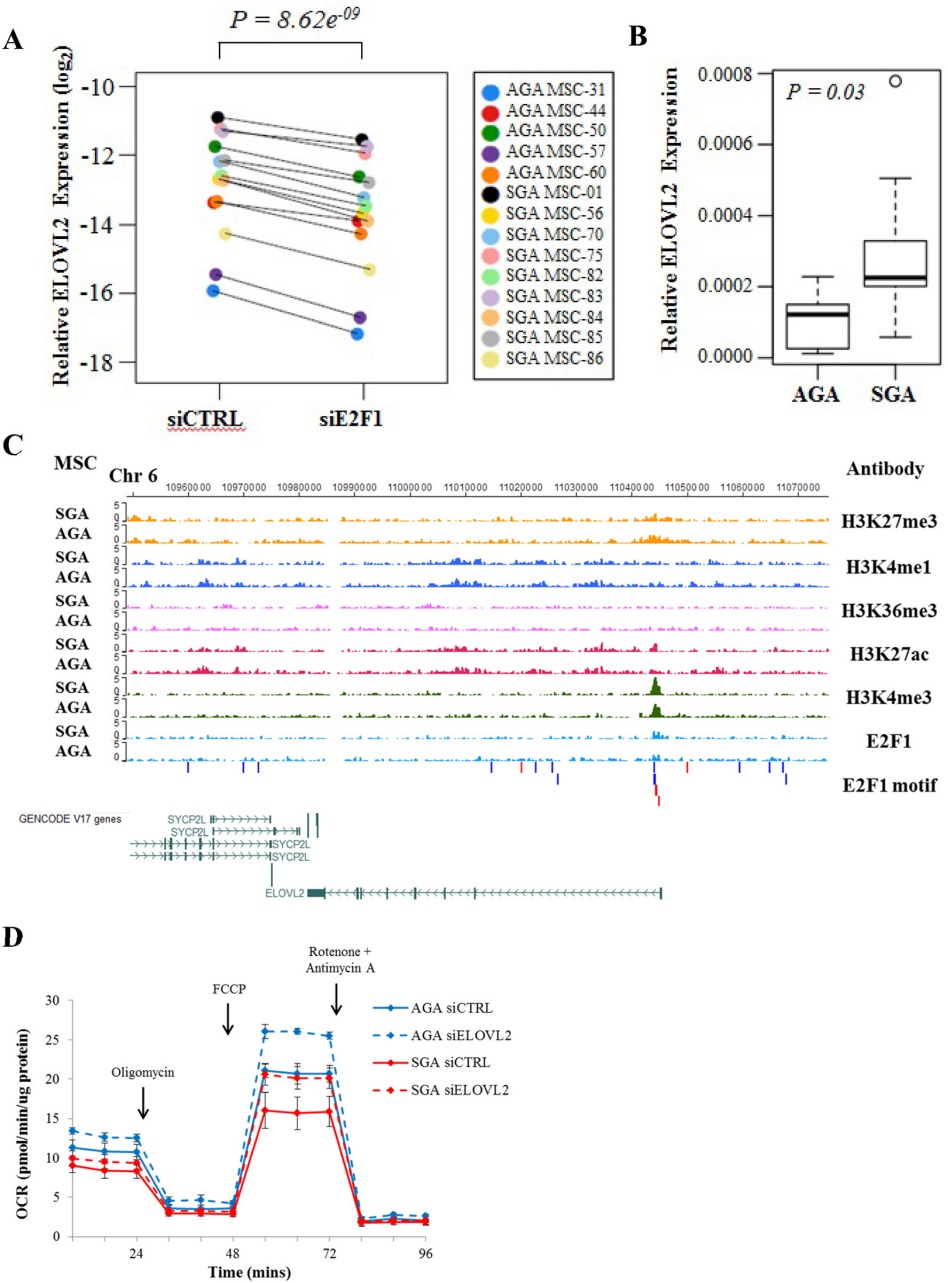
E2F1 directly activates the expression of ELOVL2 which mediates mitochondrial respiration. (A) Paired dot plot demonstrating the relative expression of ELOVL2 before and after E2F1 knockdown in 14 derived lines. Each dot represents the normalized expression of ELOVL2 gene against that of the β-actin gene. Paired colored dots indicate individual cell lines prior to and after E2F1 depletion. The data represent mean of at least 3 independent experiments. The p-value was calculated using a paired t-test. (B) Boxplot showing the relative ELOVL2 mRNA expression in 9 SGA and 5 AGA lines. Gene expression was measured by real-time RT-qPCR and ELOVL2 expression levels were normalized against those of β-actin. The data represent mean ± SEM of at least 3 independent experiments. The p-value was calculated using an unpaired t-test. (C) The screenshot shows genome browser tracks for ChIP-seq of H3K27me3, H3K4me1, H3K36me3, H3K27ac, H3K4me3 and E2F1 (tracks in orange, dark blue, pink, magenta, green and light blue, respectively) around the ELOVL2 gene in SGA- and AGA-derived MSCs. E2F1 motifs are marked by red and blue rectangular blocks indicating a forward or reverse match with respect to the reference genome. Genes in the vicinity are indicated below the tracks. (D) After siRNA transfection, mitochondrial OCR was measured in the absence and presence of drug stimulation in 6 representative MSC lines (MSC-01, MSC-56, MSC-75, MSC-44, MSC-57 and MSC-60). Red and blue lines refer to SGA- and AGA-derived MSC groups, respectively, while solid and dotted lines refer to the OCR traces before and after ELOVL2 knockdown, respectively. Results represent mean ± SEM of at least 3 independent experiments.

Next, we then sought to identify and characterize genes with important metabolic functions, whose expression is modulated by E2F1 transcriptional activity. From our E2F1 knockdown RNA-seq study, we identified ELOVL2 as a candidate gene. By performing RT-qPCR in 14 MSC lines, we validated that the suppression of E2F1 expression significantly diminished the ELOVL2 transcript level (Fig 5A). Our genome-wide expression profiling of umbilical cord-derived MSCs also revealed that ELOVL2 was overexpressed in the SGA-isolated MSCs and we further confirmed the disparate expression in more cell lines (n = 9 SGA, n = 5 AGA, Fig 5B). However, as there is currently no good antibody available to enable ELOVL2 protein detection, we could not validate the ELOVL2 overexpression at the protein level. The promoter of the ELOVL2 gene was bound by E2F1 and associated with several histone marks such as H3K27ac, H3K4me3 and H3K27me3 (Fig 5C). Interestingly, despite similar E2F1 occupancy, we observed enhanced H3K27ac and H3K4me3 modifications in the SGA-derived MSCs with correspondingly lesser H3K27me3 marks that may explain the elevated ELOVL2 expression in these cells. As shown in Fig 5D, the loss of ELOVL2 resulted in significant increase in cellular oxygen consumption and this resembles the results from the transient knockdown of E2F1 (Fig 2C). Taken together, our results suggest that ELOVL2 is a novel direct target of E2F1 and mediates a metabolic switch via regulating mitochondrial respiration.

### ELOVL2-regulation of mitochondrial genes via DHA

**Fig 6:**
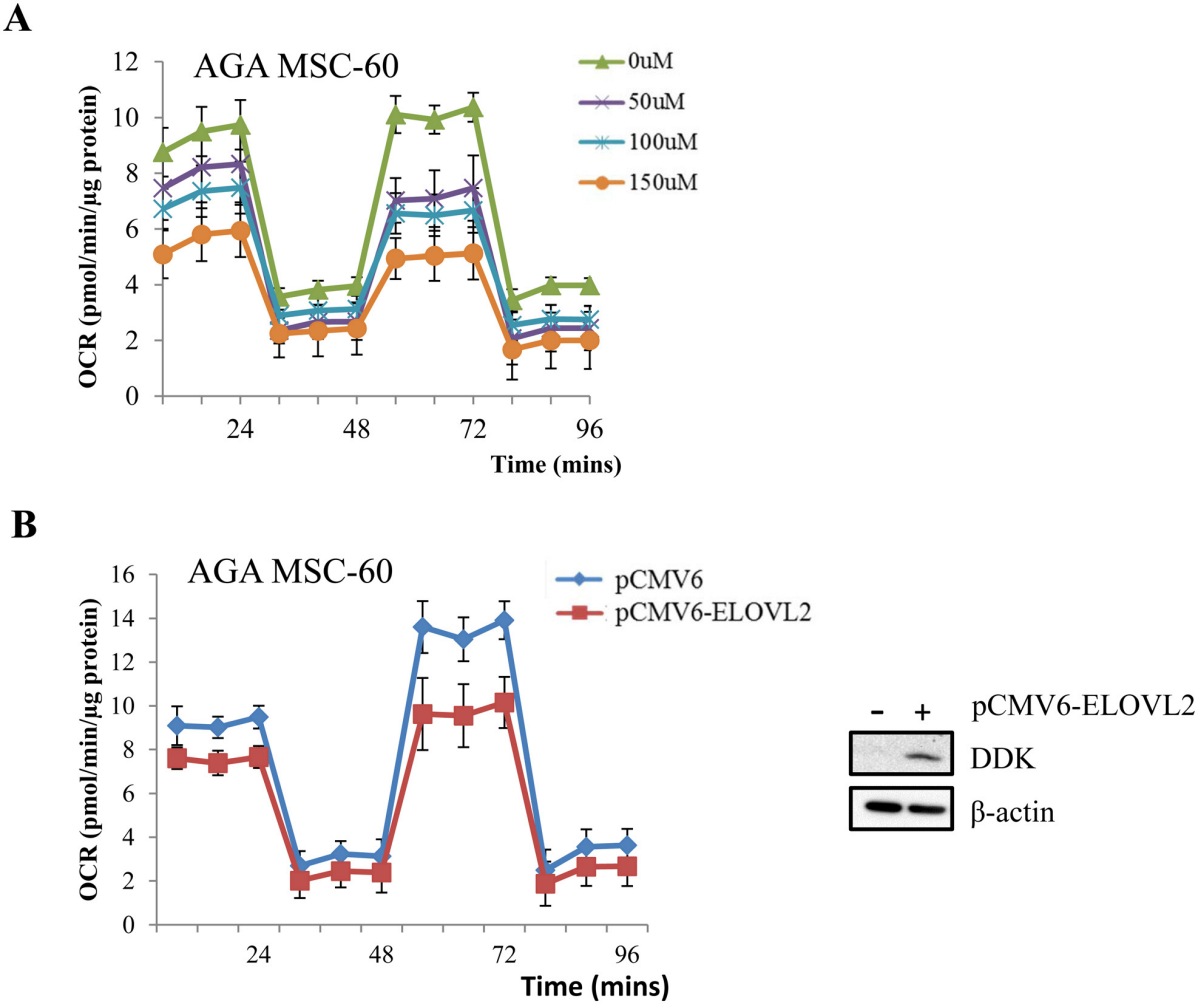
ELOVL2 controls cellular respiration possibly via DHA. (A) Upon treatment of increasing DHA concentration for 24 hrs, mitochondrial OCR was measured in a representative AGA derived MSC line (MSC-60). Results represent mean ± SEM of at least 3 independent experiments. (B) Assessment of mitochondrial OCR in a representative AGA derived MSC line (MSC-60) before and after ELOVL2 overexpression, respectively. Results represent mean ± SEM of at least 3 independent experiments. Top right: Representative western blot image before and after ELOVL2 overexpression in MSCs.

ELOVL2 is known for catalyzing the elongation of 22-carbon PUFAs to 24-carbon precursors necessary for DHA formation. Upon increasing dosage of DHA, the MSC respiratory capacity was significantly reduced (Fig 6A and S9A Fig) and this mirrors the effect of enhanced ELOVL2 expression (Fig 6B and S9B Fig), suggesting that ELOVL2 may control cellular respiration through the endogenous synthesis of DHA. In order to identify putative DEGs directly involved in mitochondrial respiration pathways, we mined the extensively curated database, MitoCarta2.0 [16]. We found 11 out of our previously identified DEGs within the database, among which LYRM5 (LYR motif-containing protein 5) and MAOA (monoamine oxidase A) gene expression increased upon DHA stimulation (S10 Fig). LYRM5 is required for the assembly of the core oxidative phosphorylation complex I [28] and its expression change may perturb the mitochondrial redox balance. Increased MAOA expression induces oxidative stress, damages the mitochondria and subdues respiration [29], which is also in line with our DHA-treated metabolic data (Fig 6A and S9A Fig). Thus, our data suggest that E2F1 and ELOVL2 may systematically fine-tune the cellular respiration through mitochondrial-related genes such as LYRM5 and MAOA.

## Discussion

Factors present at various stages during the intrauterine development are able to induce changes in fetal cells leading to changes in cellular metabolism. For example, prenatal maternal protein restriction has been shown to induce rapid proliferation of rat offspring preadipocytes [30] and our previous data from human Warton’s jelly-derived MSCs showed differential basal proliferation rates from SGA and AGA groups [7]. This increased potential for cellular proliferation may play a role in the phase of rapid catch-up growth in infancy that follows growth retardation and in the deposition of fat which has an important evolved role in protecting the programmed infant from the effects of predicted malnutrition. Insulin hypersensitive growth-restricted infants with prior exposure to lipid-rich breast milk would confer a switch to the protective state of insulin resistance once breast-feeding terminated and nutrition became more uncertain [31]. Human infants are the fattest of all mammalian species at birth and it has been suggested this is because of the high metabolic demands of the human brain and the adiposity of growth restricted infants would have been a relative protection against nutritional compromise at the time of weaning [32]. Rapidly dividing cells also utilize more glucose thereby increasing their glycolytic rate with concomitant decrease in mitochondrial oxidative phosphorylation [23]. This is reminiscent of the Warburg effect, which describes the utilization of aerobic glycolysis in highly proliferative cancer cells [33, 34]. Our MSCs taken from the SGA group of newborns show a similar metabolic profile, but Wharton’s jelly-derived MSCs are generally not transformed and hence, it remains questionable what the molecular causes are for the increased proliferation and decreased oxygen consumption rates of MSCs isolated from growth-restricted individuals.

**Fig 7:**
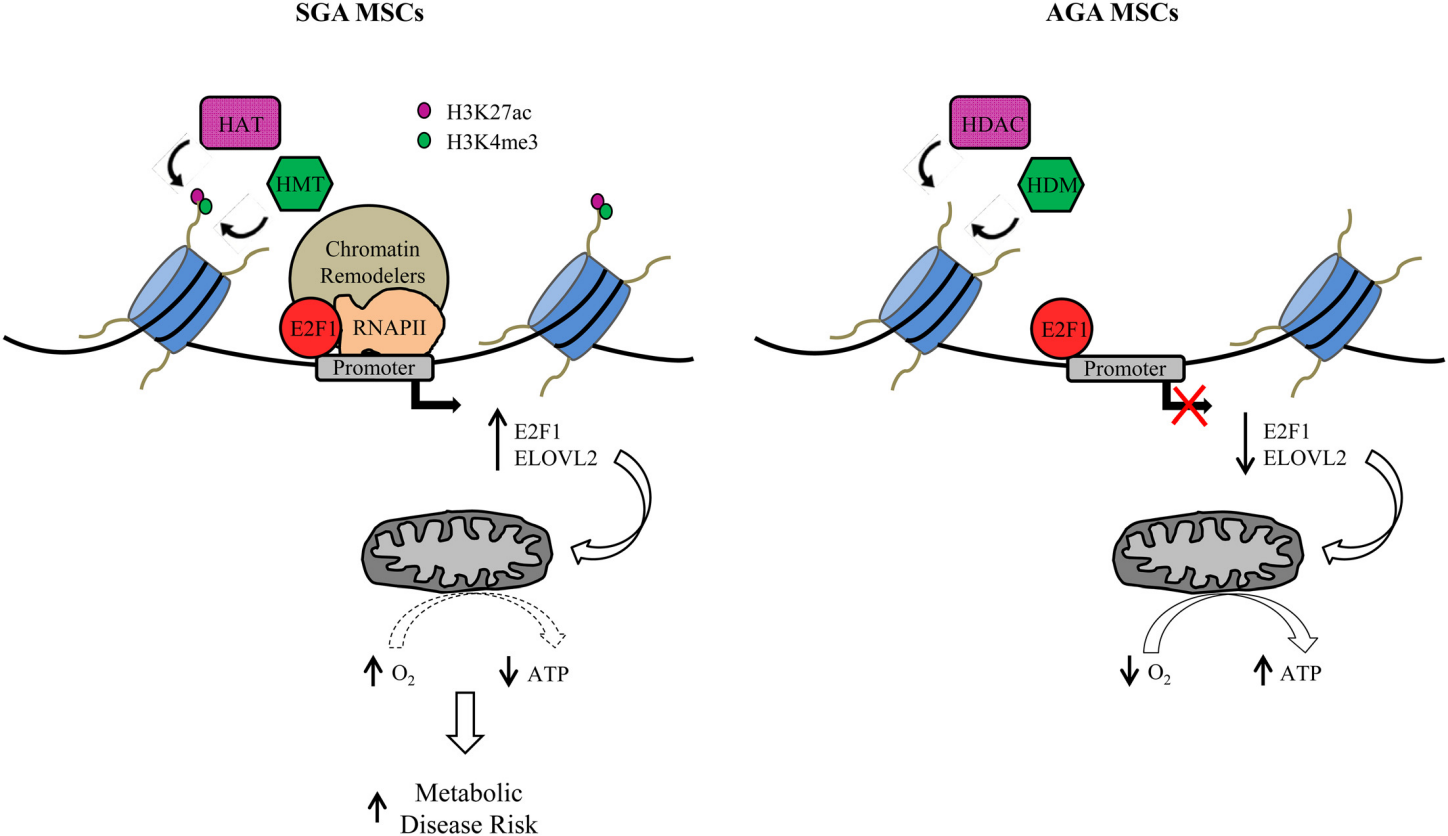
Illustration depicting the E2F1-directed transcriptional regulation of DEGs whose expressions are elevated in MSCs isolated from SGA neonates. Illustrations depicting the E2F1 directed transcriptional regulation of DEGs whose expressions are elevated in MSCs isolated from SGA neonates. Left: In the SGA-derived MSCs, E2F1 activates ELOVL2 expression via an open chromatin structure harboring histone H3K27ac and H3K4me3 marks, and resulting in impaired mitochondrial oxygen usage which may contribute to the development of metabolic health compromise in later life. Right: In AGA-derived MSCs, although E2F1 primes the promoter, epigenetic modulators are not recruited to activate the transcriptional machinery at DEGs and, thus, mitochondrial homeostasis is unperturbed. RNAPII: RNA polymerase II; HAT: Histone acetyltransferase; HMT: Histone methyltransferase; HDAC: Histone deacetylase; HDM: Histone demethylase.

Our studies show that cell cycle related pathways were found to be enriched in the SGA transcriptome with E2F1 being most prominent (Fig 1C) and that E2F1 depletion in WJ-MSCs led to changes in mitochondrial oxidative respiration (Fig 2C). Cell cycle regulators including E2F1 have previously been shown to regulate metabolism in cancer cells [23]. In particular, E2F1 was reported to suppress oxidative metabolism by acting as a metabolic switch changing from mitochondrial respiration to increased glycolysis [35]. One mechanism of how E2F1 can enhance glycolysis is the suppression of the protein deacetylase enzyme SIRT6 in prostate- and bladder cancer cells [36]. The same report revealed that E2F1 itself is under the regulation of epigenetic modifiers such as HDAC1. In our study with non-transformed Wharton’s jelly-derived MSCs, we triangulated RNA-seq expression with E2F1 siRNA knockdown and histone ChIP-seq to identify novel gene candidates regulated by E2F1 and associated epigenetic pathways. We report that E2F1 is preferentially found at the promoter of SGA-upregulated genes among the DEGs. However, interestingly, we did not observe any significant difference in the E2F1 binding strengths between the SGA and AGA groups of MSC isolates despite the higher expression levels of E2F1 and its dependent downstream gene targets in the SGA group. Instead, we noticed enhanced activating histone marks, H3K27ac and H3K4me3, at these SGA-upregulated genes in the SGA-isolated MSCs compared to their AGA counterparts (Fig 4C). Recently, a few groups have highlighted the predictive power of transcription factor binding profiles on histone modifications and subsequently targeted gene expression [37, 38], suggesting the dependency of histone modifications on transcription factor recruitment to DNA genomic regions. Herein, we postulate that E2F1 primes the promoter region of SGA-upregulated genes for potential recruitment of epigenetic modifiers leading to altered H3K27ac and H3K4me3 levels allowing gene activation (Fig 7), as we observed differential histone marks only at the gene TSS with existing E2F1 binding (see S8 Fig). However, the selective spatial recruitment of histone modifiers to these genes in the SGA but not in the AGA group of MSCs still remains to be explored and we postulate that cofactors of E2F1 may play a role in mediating such specificity. More work involving histone deacetylase and demethylase pathways needs to be conducted to further substantiate the direct epigenetic impact of H3K27ac and H3K4me3 marks on this group of associated DEGs. Notably, we also observed stronger H3K4me3 intensity at the E2F1 promoter (see S11 Fig) and this may explain the substantial increase of E2F1 expression in SGA-derived MSCs (Figs 1B, 2A and 2B).

In addition to promotion of cell cycle progression, there may be biological consequences arising from E2F1 overexpression in SGA newborns. Our findings suggest a novel pathway showing that ELOVL2 is a gene directly regulated by E2F1 (Fig 5A) and overexpressed in the SGA group of MSCs (Fig 5B). Depletion of ELOVL2 (Fig 5D) led to similar mitochondrial OCR changes as siE2F1 (Fig 2C) and ELOVL2 overexpression was able to reverse the metabolic phenotype (Fig 6B and S9B Fig). ELOVL2 is known to catalyze the endogenous elongation of PUFAs with 22 carbons resulting in the formation of DHA [14, 15] which is necessary for normal fetal development [39]. The endogenous docosapentaenoic acid (DPA) to DHA conversion step has been reported earlier to be compromised in infants with intrauterine growth restriction [40]. In addition, the DHA deficit in growth restricted fetuses may also be a consequence of maternal diets low in lipids [41]. Thus, the increased expression of ELOVL2 may seem to serve as a compensatory mechanism to supplement growth restricted infants with DHA. However, since ELOVL2 knockout mice were found to be resistant to diet-induced weight gain [15], enhancing ELOVL2 expression not only stimulates lipid synthesis but also results in weight gain. Therefore, higher ELOVL2 expression may be a risk factor for the development of obesity and associated co-morbidities for individuals with prior pre-natal SGA condition. Nonetheless, more work has to be done to confirm the increase in DHA levels upon ELOVL2 overexpression in our MSC lines.

Biomarker hypotheses for early detection of SGA have previously been made and were related to aberrant expression and function of metabolic enzymes such as glycogen phosphorylase isoenzyme BB which was found to be increased in SGA pregnancies [42] or decreased irisin levels in cord blood from SGA children [43]. The E2F1-ELOVL2-DHA axis discovered in our study of Wharton’s jelly-derived MSCs taken from growth restricted newborns has not been described before. Furthermore, our findings provided insights into the potential dynamics of metabolic respiration by modulating the expression of crucial mitochondrial genes such as LYRM5 and MAOA. It may constitute a novel pathway for targeted interventions at a very early stage in life before the onset of overt metabolic disease.

## Conclusions

We discovered ELOVL2 as a gene regulated by E2F1 in the context of fetal growth restriction. Expression of both genes was found up-regulated in Wharton’s jelly derived MSCs taken from SGA infants and we further demonstrated that both genes are associated with specific histone marks promoting their expression. The E2F1-ELOVL2 pathway suppresses mitochondrial respiration which can be viewed as a hallmark of increased metabolic disease risk.

## Supporting Information

S1 Fig Gene expression validated by RT-qPCR correlates well with RNA-seq datasets. Scatterplot of expression level of 28 transcripts measured by RT-qPCR versus basal whole transcriptomic RNA-seq in 6 representative MSC lines (MSC-01, MSC-56, MSC-75, MSC-44, MSC-57 and MSC-60).
(TIF)

S2 Fig E2F1 transient knockdown efficiency in MSCs. (A) Bars showing the knockdown efficiency of siE2F1 when compared to siCTRL in 6 MSC lines (MSC-01, MSC-56, MSC-75, MSC-44, MSC-57 and MSC-60). Total mRNA expression was quantified by real-time RT-qPCR. E2F1 expression levels were normalized against those of β-actin before comparing siE2F1 condition relative to that of siCTRL. The data represent mean ± SEM of at least 3 independent experiments (* p<0.001). (B) E2F1 and β-actin protein expression levels after transfection of siCTRL or siE2F1 in a representative MSC line, MSC-56.
(TIF)

S3 Fig E2F1 binds predominantly at gene TSS in Wharton’s jelly MSCs. (A) Percentage of E2F1 binding in 6 MSC lines at different genomic locations relative to nearest transcription units from the gencode V19 database. (B) Distribution of E2F1 occupancy within 3 kb up- and downstream of gene TSS.
(TIF)

S4 Fig Tables showing the number of genes observed in different subcategories. (A) Number of genes with differential basal expression between the derived groups of cell lines and (B) gene expression changes upon E2F1 knockdown (KD) or (C) both, with their respective E2F1 binding strength at the gene TSS. Categories of “S > A”, “S < A”, “S = A” and “UN” represent DEGs with higher basal expression in SGA, DEGs with higher basal expression in AGA, expressed genes excluding DEGs and unexpressed genes, respectively. Categories of “↓”, “↑” and “-” represent genes whose expressions were downregulated, upregulated or remained unchanged upon siE2F1 treatment, respectively. A p-value of <0.005 was obtained using the Fisher’s Exact Test on the subcategory with the following combined attributes: basal expression of “S > A”, KD of “↓” and E2F1 binding of “Moderate” and “High”.
(TIF)

S5 Fig De novo motif discovery by HOMER shows E2F1 as the most enriched transcription factor motif in Wharton’s jelly MSCs. Top 500 binding sites from our E2F1 ChIP-seq libraries were fed into HOMER for de novo motif discovery. The top panel shows the de novo motif analysis output while the bottom panel shows one of the E2F1 motifs from HOMER’s known motif database.
(TIF)

S6 Fig Gene Ontology analyses for SGA-upregulated DEGs. Metacore GO of DEGs which have higher basal expression in SGA compared to AGA-derived MSCs. Top and bottom tables are GO results from DEGs with high and moderate fold change differences between SGA and AGA groups. Tables show the top ten biological processes ranked by FDR.
(TIF)

S7 Fig Differential enrichment of E2F1 motif between SGA and AGA groups of cell lines at promoters of DEGs with higher expression in SGA-derived MSCs. (A) Top 10 transcription factor family motifs found by HOMER to be differentially enriched at promoters of DEGs which are upregulated in SGA-isolated MSCs compared to promoters of DEGs which are upregulated in AGA-isolated MSCs. (B) Vertical bar plot depicting the proportion of DEGs containing E2F1 binding from multiple publicly available ChIP-seq data sets belonging to the ENCODE project. S > A: DEGs with higher expression in SGA-derived MSCs; S < A: DEGs with higher expression in AGA-derived MSCs. The p-value was calculated using a Fisher’s Exact Test.
(TIF)

S8 Fig Only E2F1-bound DEGs have significantly higher H3K27ac and H3K4me3 modifications in SGA-derived vs AGA-derived MSCs. Scatterplots illustrating ChIP-seq intensities of H3K27ac (left) and H3K4me3 (right) between SGA and AGA-derived MSCs at SGA-upregulated DEGs. Top: DEGs with E2F1 binding at the promoter; bottom: DEGs with negligible E2F1 binding at the promoter. The p-values were calculated using a two-tailed binomial test.
(TIF)

S9 Fig ELOVL2 controls cellular respiration possibly via DHA in additional AGA and SGA MSC lines. (A) Upon treatment with increasing DHA concentration for 24 hrs, mitochondrial OCR was measured in 3 additional MSC lines (MSC-44, MSC-56 and MSC-70). Results represent mean ± SEM of at least 3 independent experiments. (B) Assessment of mitochondrial OCR in 2 additional MSC lines (MSC-70 and MSC-56) before and after ELOVL2 overexpression, respectively. Results represent mean ± SEM of at least 3 independent experiments.
(TIF)

S10 Fig Elevated DHA levels lead to concentration-dependent increase in gene expression of LYRM5 and MAOA which are important in maintaining the homeostasis of mitochondrial respiration. Relative expression levels of (A) LYRM5 and (B) MAOA in the presence of DHA stimulation prior and after DHA treatment in 3 representative MSC lines. The data represent mean ± SEM of at least 3 independent experiments. Unpaired t-test was calculated between 0 μM and 150 μM DHA treated conditions, * p < 0.05.
(TIF)

S11 Fig ChIP-seq profiles of E2F1 and histone marks, as well as E2F1 motif in the vicinity of the E2F1 gene. The screenshot shows the genome browser tracks for ChIP-seq of H3K27me3, H3K4me1, H3K36me3, H3K27ac, H3K4me3 and E2F1 (tracks in orange, dark blue, pink, magenta, green and light blue, respectively) around the E2F1 gene in SGA- and AGA-derived MSCs. E2F1 motifs are marked by red and blue rectangular indicating forward or reverse match with respect to the reference genome. Genes in the vicinity are indicated below the tracks.
(TIF)

S1 Table Sequences of gene-specific PCR primers and small interfering RNA oligonucleotides. (XLSX)
