# Supplementary figures and images for: E2F1 Orchestrates Transcriptomics and Oxidative Metabolism in Wharton’s Jelly-Derived Mesenchymal Stem Cells from Growth-Restricted Infants
Source: PLoS One. 2016 Sep 15;11(9):e0163035. doi: 10.1371/journal.pone.0163035 (PMC5025055; doi:10.1371/journal.pone.0163035)

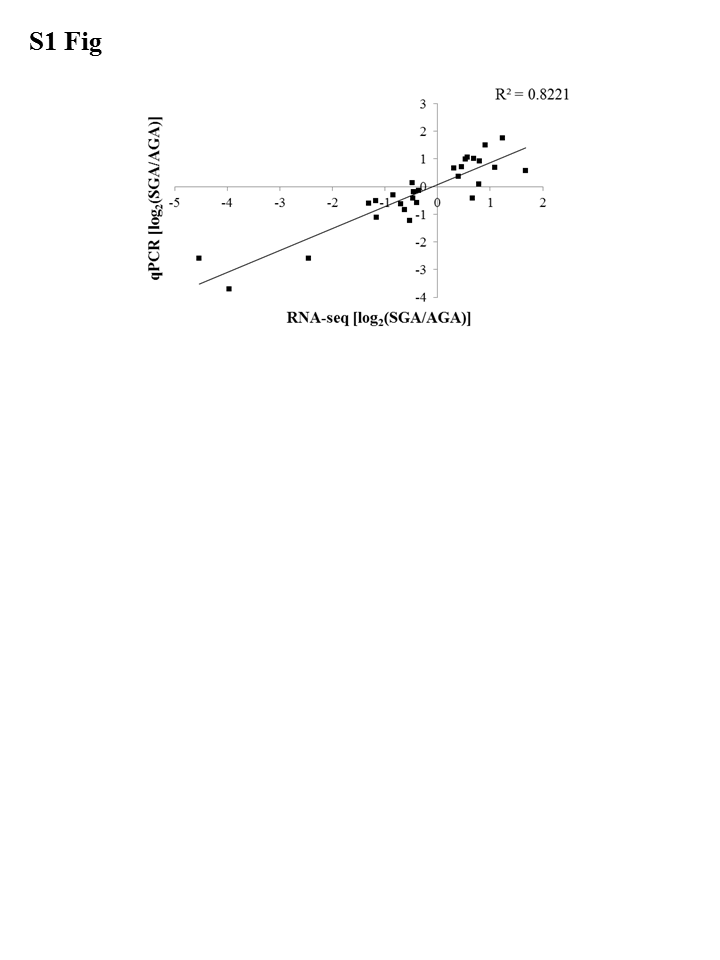

Supplement: S1 Fig — Scatterplot of expression level of 28 transcripts measured by RT-qPCR versus basal whole transcriptomic RNA-seq in 6 representative MSC lines (MSC-01, MSC-56, MSC-75, MSC-44, MSC-57 and MSC-60). (TIF) [file pone.0163035.s001.tif]

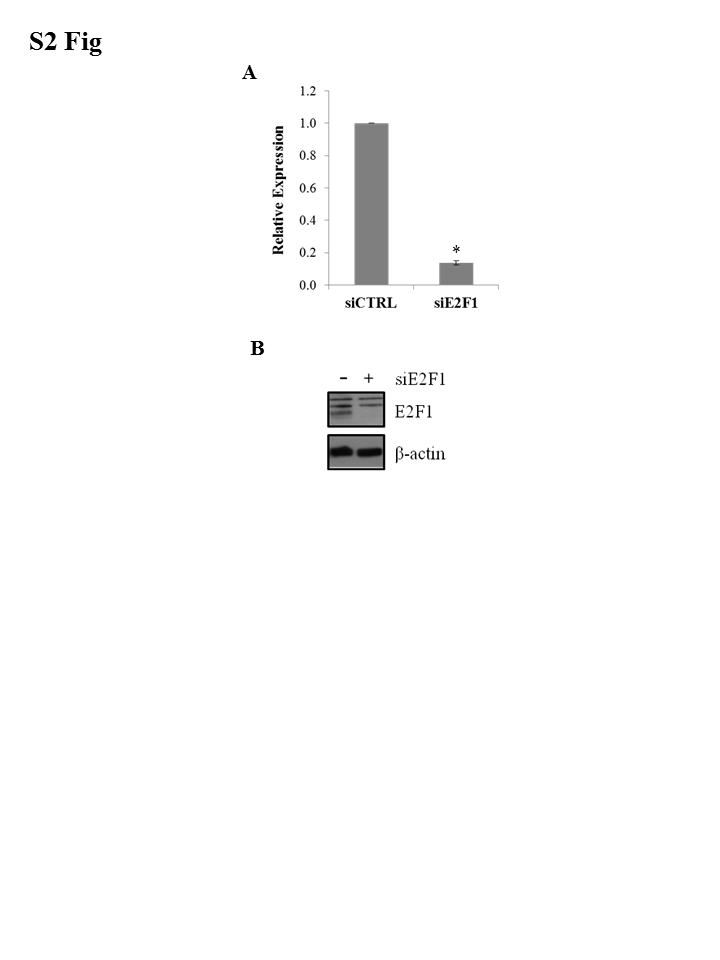

Supplement: S2 Fig — (A) Bars showing the knockdown efficiency of siE2F1 when compared to siCTRL in 6 MSC lines (MSC-01, MSC-56, MSC-75, MSC-44, MSC-57 and MSC-60). Total mRNA expression was quantified by real-time RT-qPCR. E2F1 expression levels were normalized against those of β-actin before comparing siE2F1 condition relative to that of siCTRL. The data represent mean ± SEM of at least 3 independent experiments (* p<0.001). (B) E2F1 and β-actin protein expression levels after transfection of siCTRL or siE2F1 in a representative MSC line, MSC-56. (TIF) [file pone.0163035.s002.tif]

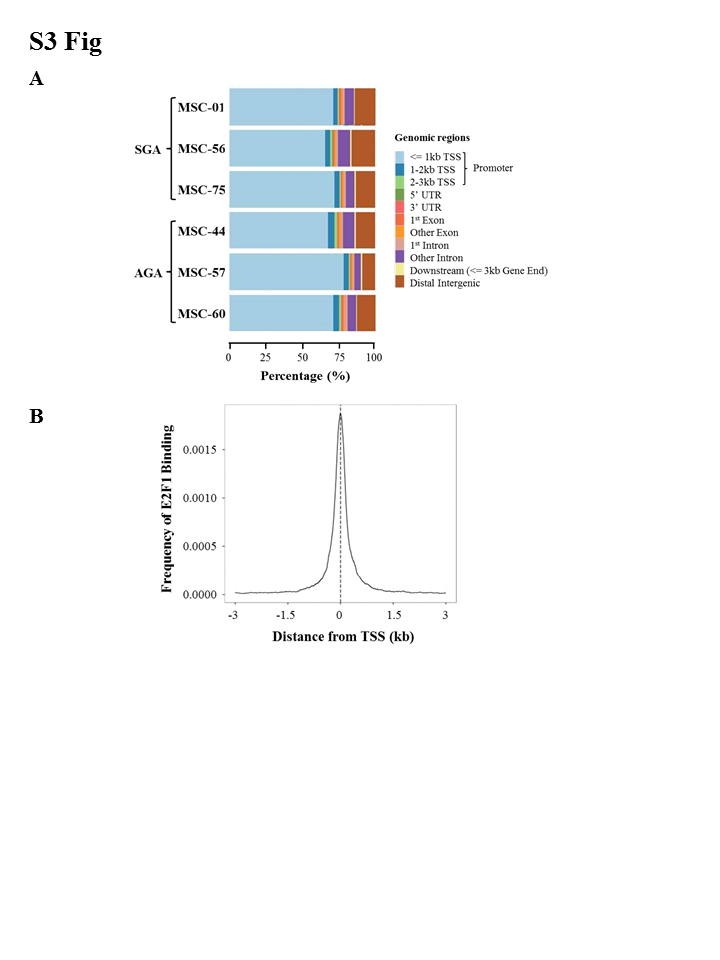

Supplement: S3 Fig — (A) Percentage of E2F1 binding in 6 MSC lines at different genomic locations relative to nearest transcription units from the gencode V19 database. (B) Distribution of E2F1 occupancy within 3 kb up- and downstream of gene TSS. (TIF) [file pone.0163035.s003.tif]

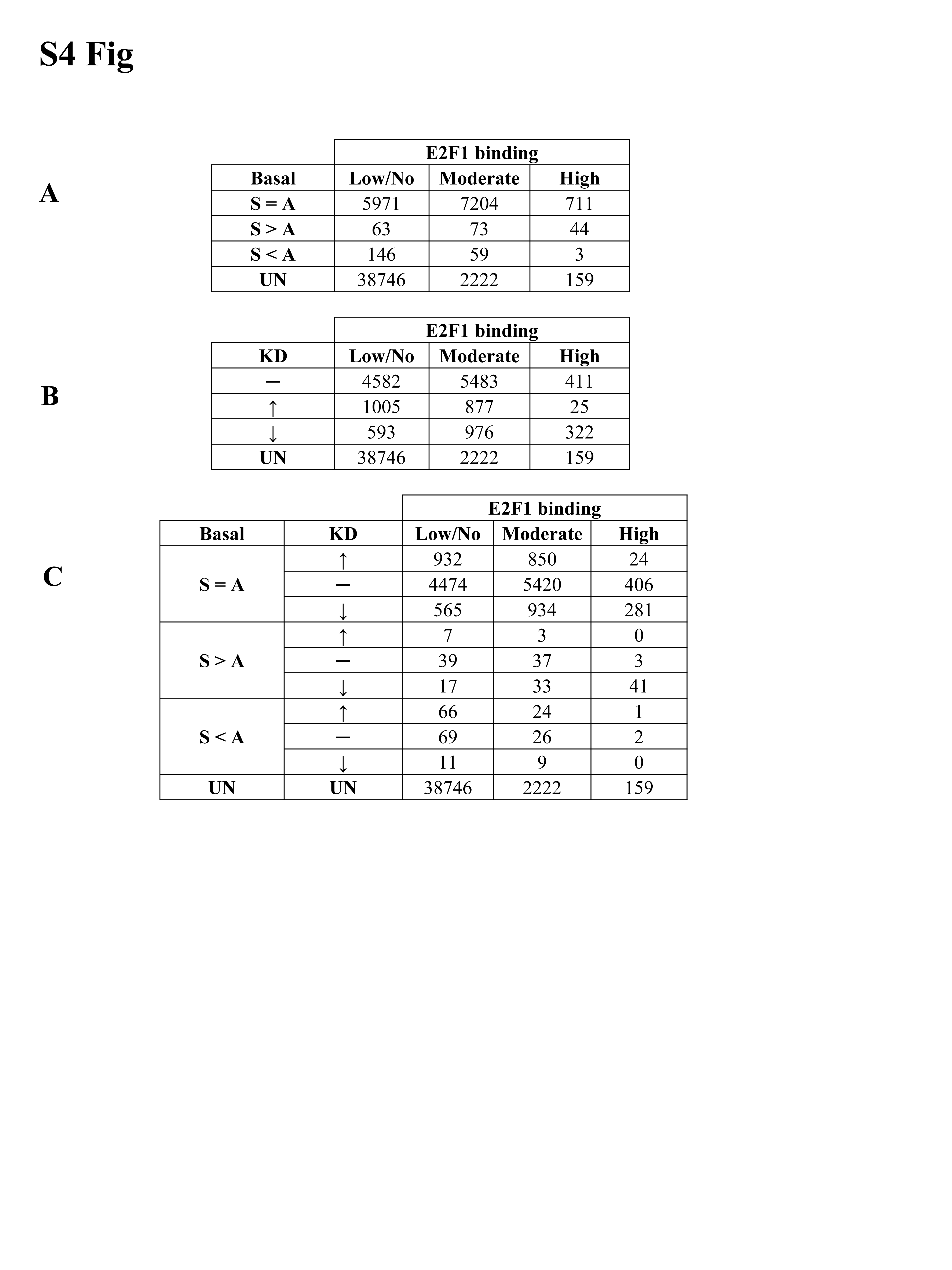

Supplement: S4 Fig — (A) Number of genes with differential basal expression between the derived groups of cell lines and (B) gene expression changes upon E2F1 knockdown (KD) or (C) both, with their respective E2F1 binding strength at the gene TSS. Categories of “S > A”, “S < A”, “S = A” and “UN” represent DEGs with higher basal expression in SGA, DEGs with higher basal expression in AGA, expressed genes excluding DEGs and unexpressed genes, respectively. Categories of “↓”, “↑” and “-” represent genes whose expressions were downregulated, upregulated or remained unchanged upon siE2F1 treatment, respectively. A p-value of <0.005 was obtained using the Fisher’s Exact Test on the subcategory with the following combined attributes: basal expression of “S > A”, KD of “↓” and E2F1 binding of “Moderate” and “High”. (TIF) [file pone.0163035.s004.tif]

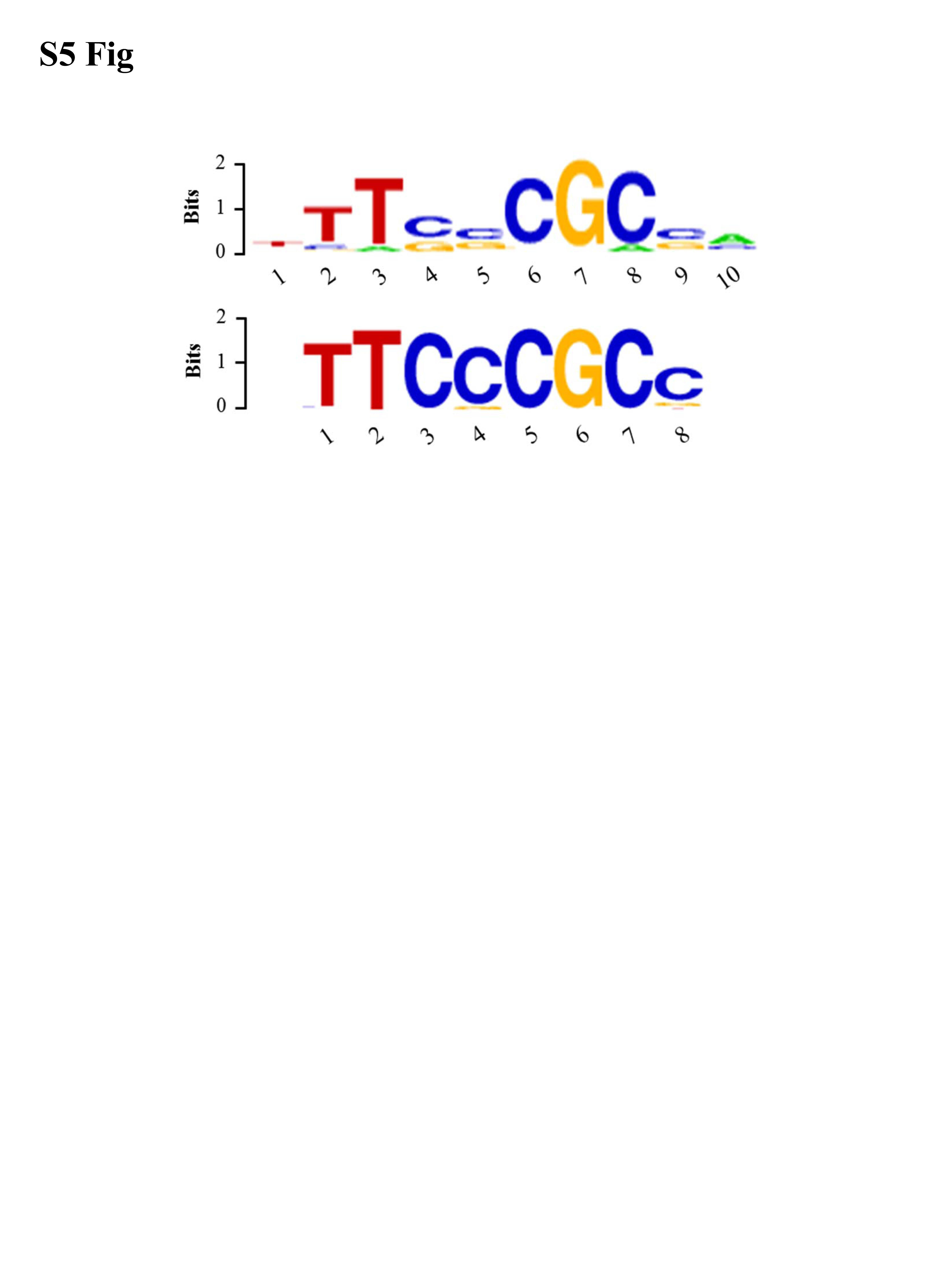

Supplement: S5 Fig — Top 500 binding sites from our E2F1 ChIP-seq libraries were fed into HOMER for de novo motif discovery. The top panel shows the de novo motif analysis output while the bottom panel shows one of the E2F1 motifs from HOMER’s known motif database. (TIF) [file pone.0163035.s005.tif]

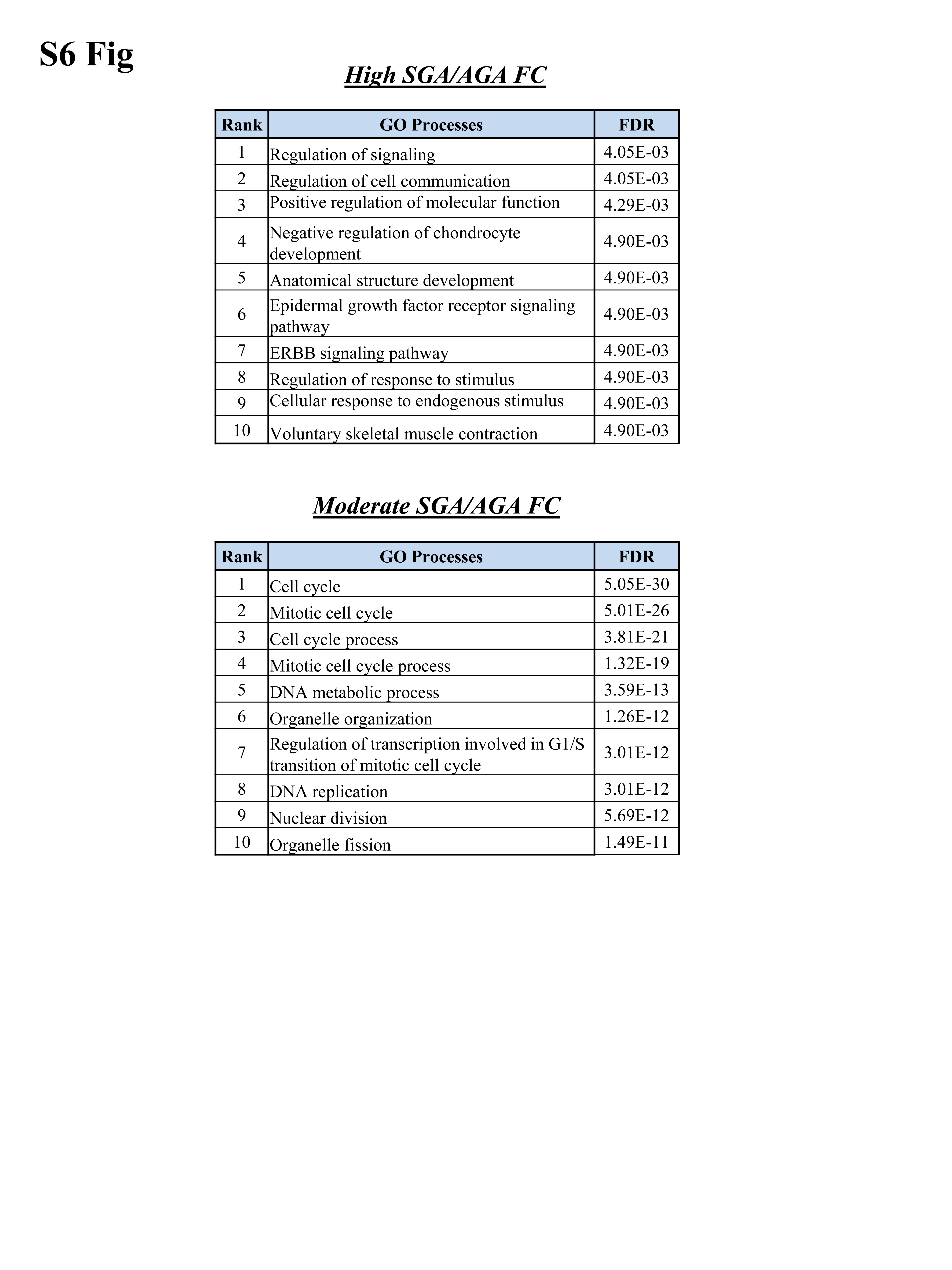

Supplement: S6 Fig — Metacore GO of DEGs which have higher basal expression in SGA compared to AGA-derived MSCs. Top and bottom tables are GO results from DEGs with high and moderate fold change differences between SGA and AGA groups. Tables show the top ten biological processes ranked by FDR. (TIF) [file pone.0163035.s006.tif]

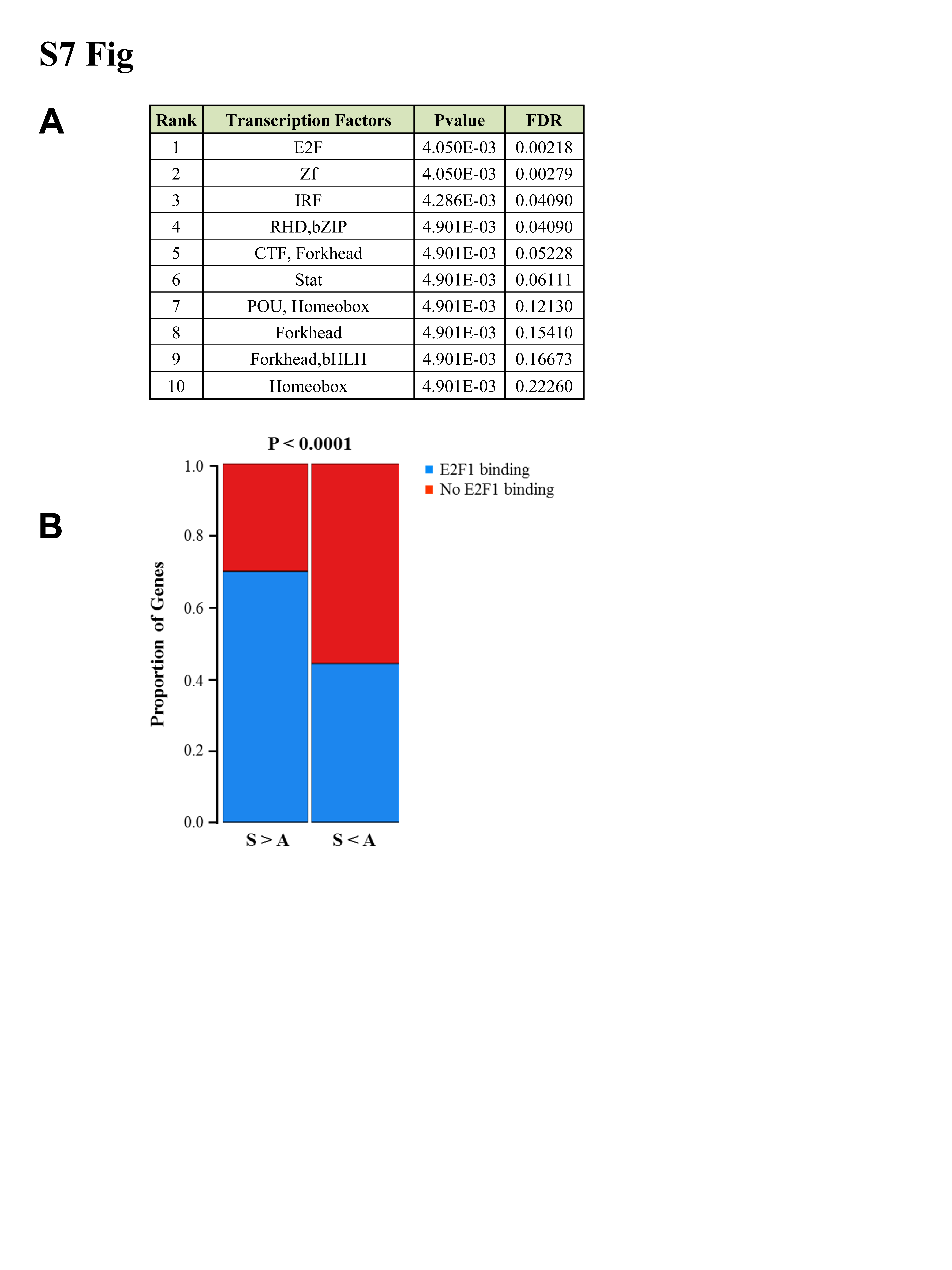

Supplement: S7 Fig — (A) Top 10 transcription factor family motifs found by HOMER to be differentially enriched at promoters of DEGs which are upregulated in SGA-isolated MSCs compared to promoters of DEGs which are upregulated in AGA-isolated MSCs. (B) Vertical bar plot depicting the proportion of DEGs containing E2F1 binding from multiple publicly available ChIP-seq data sets belonging to the ENCODE project. S > A: DEGs with higher expression in SGA-derived MSCs; S < A: DEGs with higher expression in AGA-derived MSCs. The p-value was calculated using a Fisher’s Exact Test. (TIF) [file pone.0163035.s007.tif]

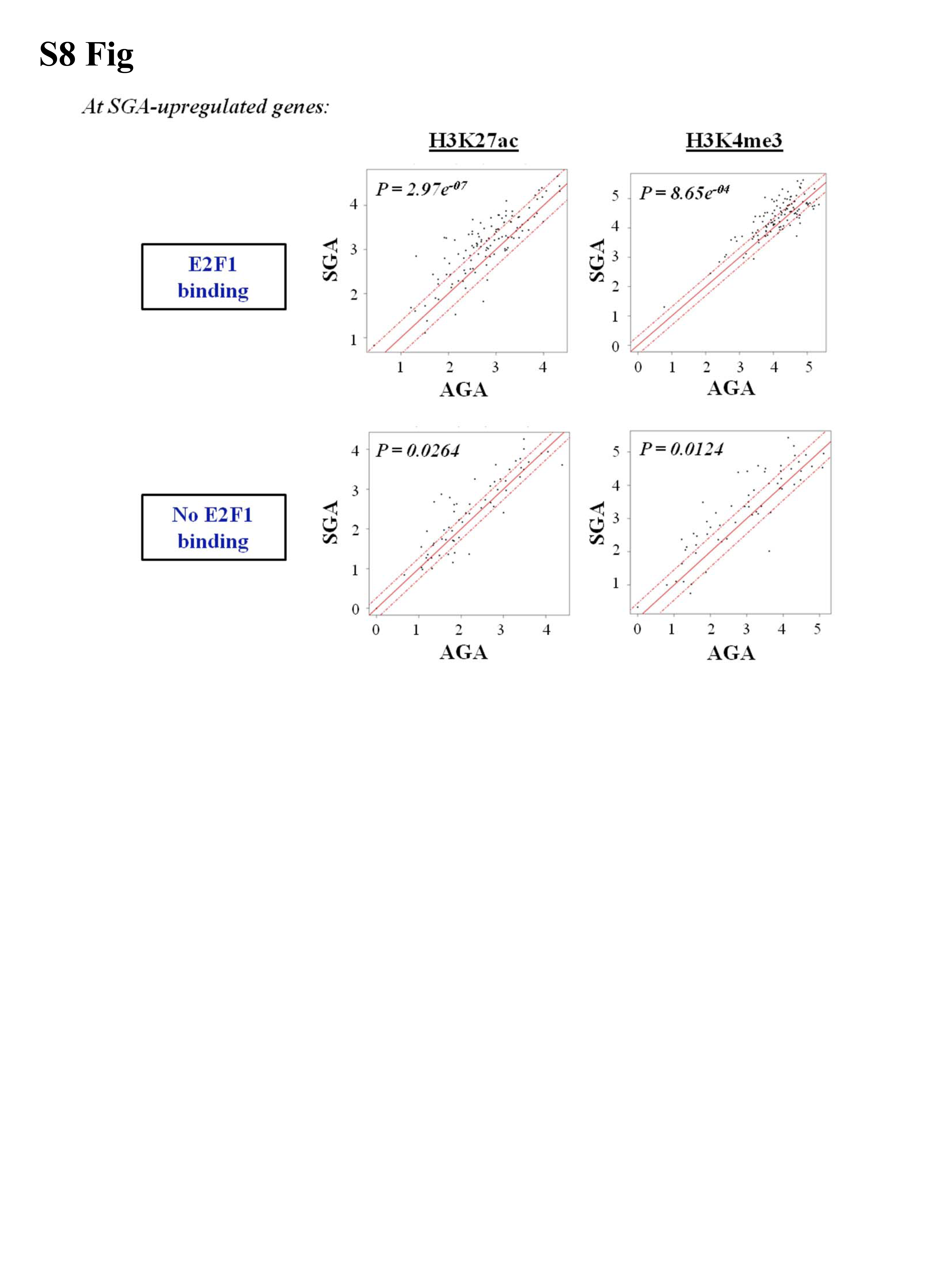

Supplement: S8 Fig — Scatterplots illustrating ChIP-seq intensities of H3K27ac (left) and H3K4me3 (right) between SGA and AGA-derived MSCs at SGA-upregulated DEGs. Top: DEGs with E2F1 binding at the promoter; bottom: DEGs with negligible E2F1 binding at the promoter. The p-values were calculated using a two-tailed binomial test. (TIF) [file pone.0163035.s008.tif]

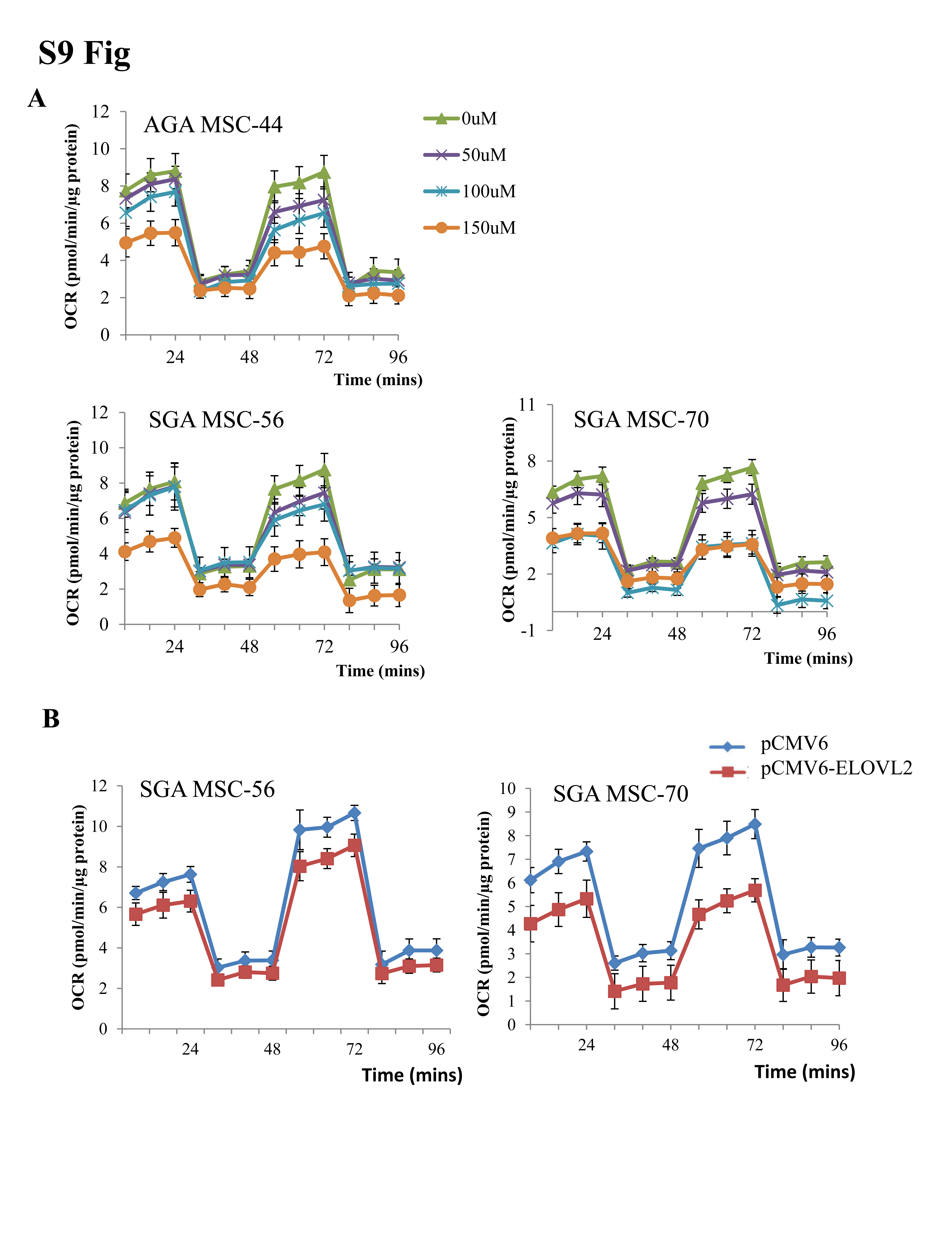

Supplement: S9 Fig — (A) Upon treatment with increasing DHA concentration for 24 hrs, mitochondrial OCR was measured in 3 additional MSC lines (MSC-44, MSC-56 and MSC-70). Results represent mean ± SEM of at least 3 independent experiments. (B) Assessment of mitochondrial OCR in 2 additional MSC lines (MSC-70 and MSC-56) before and after ELOVL2 overexpression, respectively. Results represent mean ± SEM of at least 3 independent experiments. (TIF) [file pone.0163035.s009.tif]

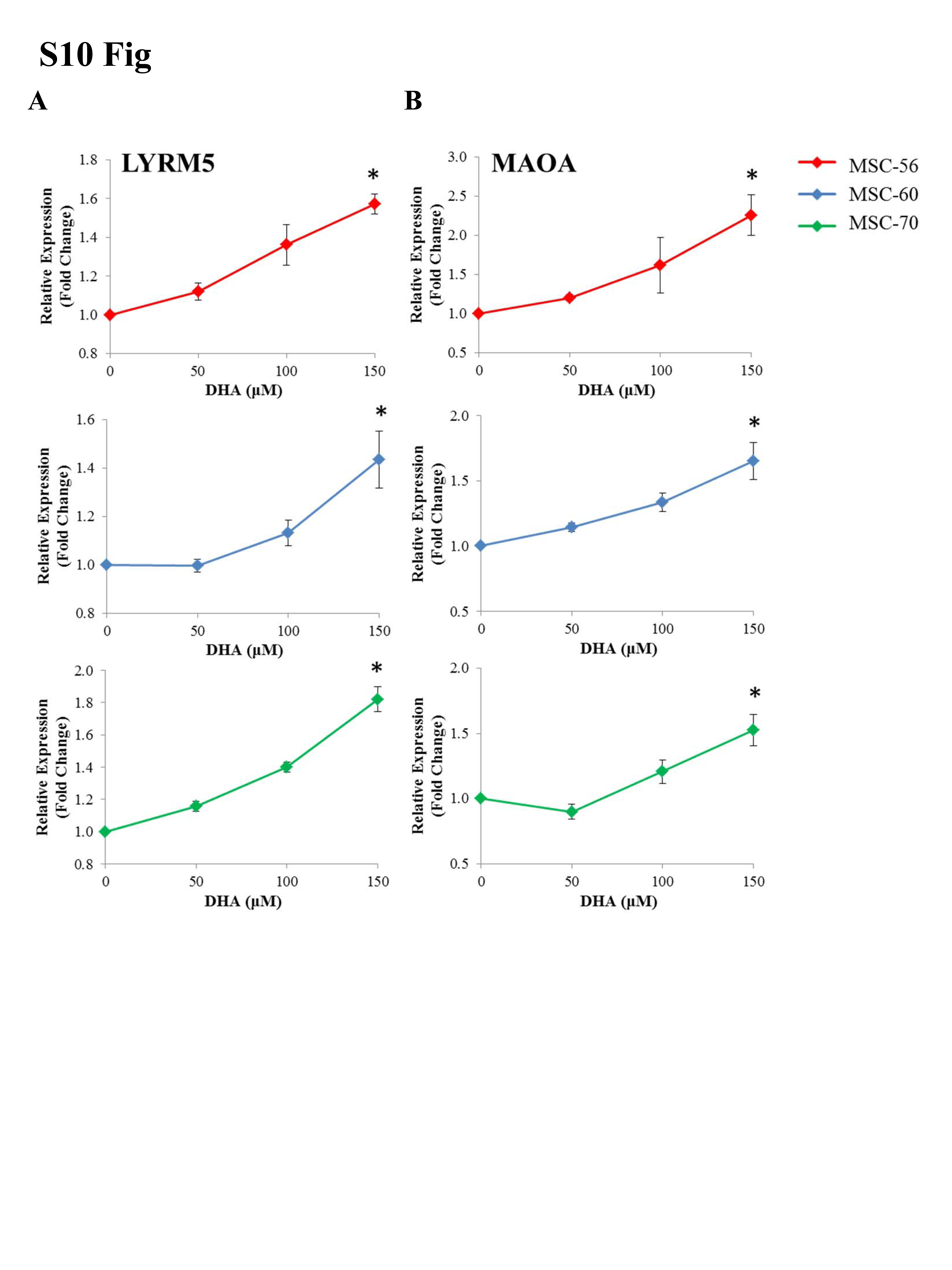

Supplement: S10 Fig — Relative expression levels of (A) LYRM5 and (B) MAOA in the presence of DHA stimulation prior and after DHA treatment in 3 representative MSC lines. The data represent mean ± SEM of at least 3 independent experiments. Unpaired t-test was calculated between 0 μM and 150 μM DHA treated conditions, * p < 0.05. (TIF) [file pone.0163035.s010.tif]

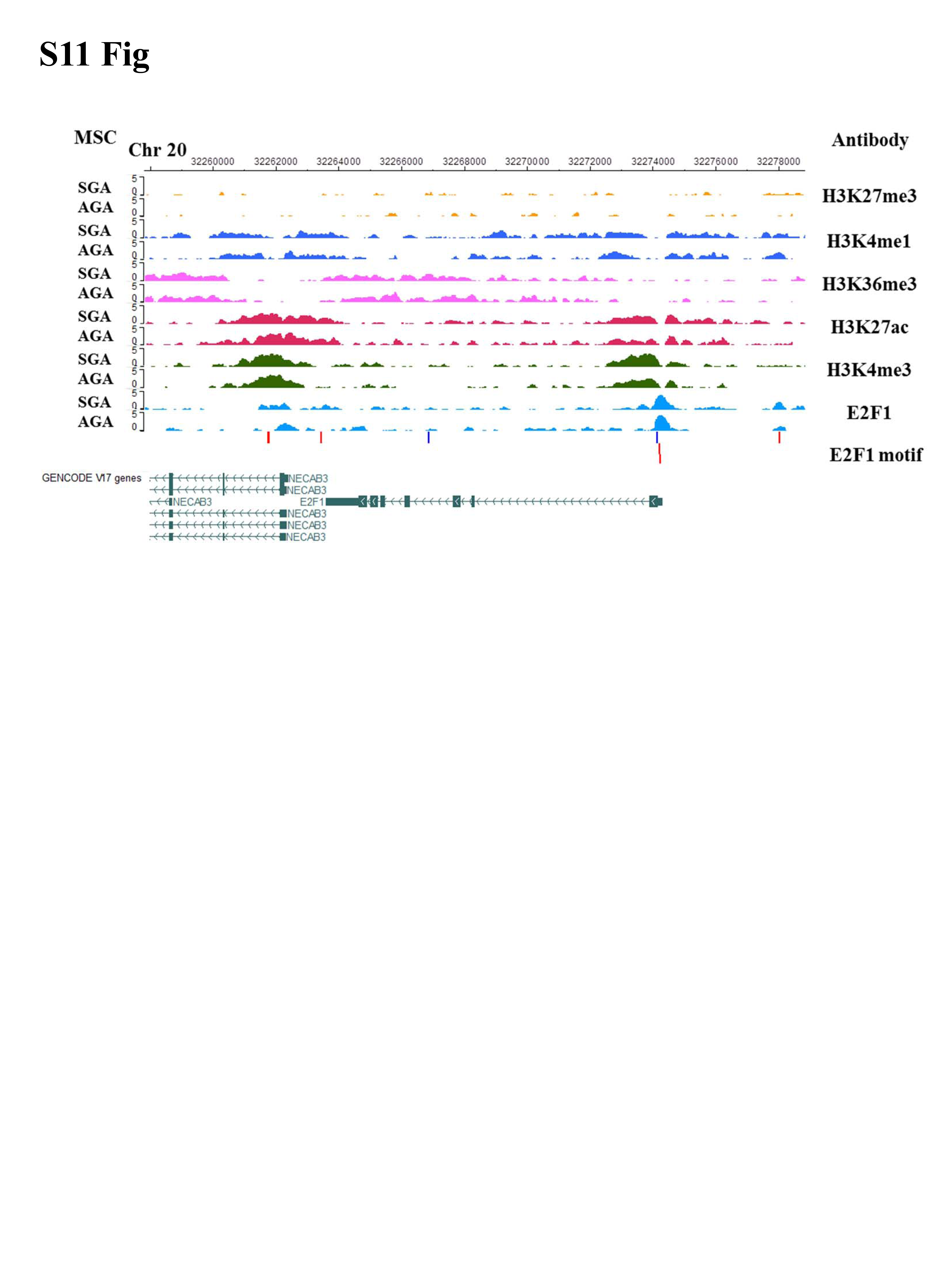

Supplement: S11 Fig — The screenshot shows the genome browser tracks for ChIP-seq of H3K27me3, H3K4me1, H3K36me3, H3K27ac, H3K4me3 and E2F1 (tracks in orange, dark blue, pink, magenta, green and light blue, respectively) around the E2F1 gene in SGA- and AGA-derived MSCs. E2F1 motifs are marked by red and blue rectangular indicating forward or reverse match with respect to the reference genome. Genes in the vicinity are indicated below the tracks. (TIF) [file pone.0163035.s011.tif]
